# Implications of Organic Dairy Management on Herd Performance and Milk Fatty Acid Profiles and Interactions with Season

**DOI:** 10.3390/foods12081589

**Published:** 2023-04-08

**Authors:** Sabrina Ormston, Nanbing Qin, Gergely Faludi, Joe Pitt, Alan W. Gordon, Katerina Theodoridou, Tianhai Yan, Sharon A. Huws, Sokratis Stergiadis

**Affiliations:** 1 Department of Animal Sciences, School of Agriculture, Policy and Development, University of Reading, Earley Gate, P.O. Box 237, Reading RG6 6EU, UK; s.ormston@pgr.reading.ac.uk (S.O.); superqnb100@sina.com (N.Q.); fg910722@gmail.com (G.F.); joepaxtonpitt@yahoo.co.uk (J.P.); 2 Department of Animal Breeding, Georgikon Campus, Institute of Animal Science, Hungarian University of Agriculture and Life Sciences, Deák Ferenc u. 16, H-8360 Keszthely, Hungary; 3 Statistical Services Branch, Agri-Food and Biosciences Institute, Newforge Lane, Belfast BT9 5PX, UK; alan.gordon@afbini.gov.uk; 4 School of Biological Sciences, Institute for Global Food Security, Queen’s University Belfast, Belfast BT9 5DL, UK; k.theodoridou@qub.ac.uk (K.T.); s.huws@qub.ac.uk (S.A.H.); 5 Livestock Production Sciences Branch, Agri-Food and Biosciences Institute, Large Park, Hillsborough BT26 6DR, UK; tianhai.yan@afbini.gov.uk

**Keywords:** dairy, efficiency, fatty acids, milk, organic

## Abstract

Interest in organic cows’ milk has increased due to the perceived superior nutritional quality and improved sustainability and animal welfare. However, there is a lack of simultaneous assessments on the influence of organic dairy practices and dietary and breed drivers on productivity, feed efficiency, health parameters, and nutritional milk quality at the herd level. This work aimed to assess the impact of organic vs. conventional management and month on milk yield and basic composition, herd feed efficiency, health parameters, and milk fatty acid (FA) composition. Milk samples (*n* = 800) were collected monthly from the bulk tanks of 67 dairy farms (26 organic and 41 conventional) between January and December 2019. Data on breed and feeding practices were gathered via farm questionnaires. The samples were analyzed for their basic composition and FA profile using Fourier transform infrared spectroscopy (FTIR) and gas chromatography (GC), respectively. The data were analyzed using a linear mixed model, repeated measures design and multivariate redundancy analysis (RDA). The conventional farms had higher yields (kg/cow per day) of milk (+7.3 kg), fat (+0.27 kg), and protein (+0.25 kg) and higher contents (g/kg milk) of protein, casein, lactose, and urea. The conventional farms produced more milk (+0.22 kg), fat (+8.6 g), and protein (+8.1 g) per kg offered dry matter (DM). The organic farms produced more milk per kg of offered non-grazing and concentrate DM offered, respectively (+0.5 kg and +1.23 kg), and fat (+20.1 g and +51 g) and protein (+17 g and +42 g). The organic milk had a higher concentration of saturated fatty acid (SFA; +14 g/kg total FA), polyunsaturated fatty acid (PUFA; +2.4 g/kg total FA), and nutritionally beneficial FA alpha linolenic acid (ALNA; +14 g/kg total FA), rumenic acid (RA; +14 g/kg total FA), and eicosapentaenoic acid (EPA; +14 g/kg total FA); the conventional milk had higher concentrations of monounsaturated FA (MUFA; +16 g/kg total FA). Although the conventional farms were more efficient in converting the overall diet into milk, fat, and protein, the organic farms showed better efficiency in converting conserved forages and concentrates into milk, fat, and protein as a result of reduced concentrate feeding. Considering the relatively small differences in the FA profiles between the systems, increased pasture intake can benefit farm sustainability without negatively impacting consumer nutrition and health.

## 1. Introduction

Organic dairy farming regulations in the UK require farms to adopt high grazing or forage intake, with reduced amounts of concentrate feed; during times when grazing is not possible or not sufficient, forage must still contribute >60% to the dry matter (DM) of the animal diets [1]. In response to the interest in organic or pasture-fed cows’ milk [2], previous studies have assessed productivity and milk parameters from organic vs. conventional [3,4,5], low-intensity vs. high-intensity (e.g., 95 vs. 37% fresh forage in the diet (proportion of offered DM) by Butler, et al. [4], and 84.3 vs. 42.6% pasture intake in DMI by Stergiadis, et al. [5], respectively), or indoor vs. outdoor farms [4,6]. Some of these studies included assessments of solids’ composition [6] and fatty acid (FA) profile [3,4,7], while others focused on animal health and reproductive efficiency [6]. Previous literature has found reduced milk yields [4,5,8] yet increased milk fat and protein content in pasture-based, organic, and low-input systems when compared with high-intensity or conventional indoor systems [5,6]. However, when Stergiadis et al. [9] compared organic, low-input systems to nonorganic, low-input systems, no difference between fat and protein content was observed [9]. Studies that have identified higher milk fat and protein concentrations in organic or low-intensity production systems have attributed this to the inclusion of breeds other than Holstein, as well as to increased grazing, which is associated with organic and low-input systems [5]. Investigations into milk FA have demonstrated lower concentrations of the nutritionally undesirable saturated fatty acids (SFA) [10] and higher concentrations of the human-health beneficial monounsaturated fatty acids (MUFA) and polyunsaturated fatty acids (PUFA) [10] in low-intensity dairy systems compared to high-intensity systems [4], and in both organic and low-input systems when compared with conventional systems [5]. Additionally, investigations into the effect of season or month have been carried out, yielding consistent results; Ellis et al. [3] found no difference in overall SFA content between conventional and organic milk, but when month was examined, the SFA content in both organic and conventional milk was significantly higher during the winter months, when cows were most likely housed indoors without access to pasture and provided with a total mixed ration. Similarly, nutritionally desirable FA, conjugated linoleic acid (CLA), and vaccenic acid (VA) were highest during summer months while cows were grazing [3]. Butler, et al. [4] also compared production intensity, demonstrating higher milk concentrations of CLA, VA, and MUFA when grazing contributed 95% of the total intake. These results have been attributed to the high alpha linolenic acid (ALNA) content of fresh herbage [3].

Feed efficiency is highly important in both organic and conventional systems from a profitability, food security, and sustainability perspective [11]. For organic and low-input systems, the efficient conversion of forage or pasture to milk is particularly important, given the higher reliance on grazing and foraging when compared to other production systems [8]. The few studies that have investigated the efficiency between these production systems have identified higher feed efficiency in conventional systems when compared with organic systems [11,12]. A meta-analysis carried out by Gaudaré et al. [11] reported a 14% decrease in herd feed efficiency (milk output/dry matter intake (DMI)) in organic systems when compared to conventional systems. Similar results have been found by Brito and Silva [12], reporting a 16% lower feed efficiency in organic Jersey cows when compared with conventional system Jersey cows. Interestingly, when concentrate efficiency was examined (milk output (kg)/DMI from concentrate (kg)), organic dairy cows were found to produce more milk per kg concentrate, probably due to the lower reliance on concentrate feed [11].

Although a few studies have compared different parameters regarding productivity and milk quality in organic production systems, the assessment of herd feed efficiency has not been performed in UK dairy systems before, with only a few studies globally [11,12]. There is also currently a lack of a simultaneous assessment of milk production and solids composition, nutritional quality (e.g., fatty acid profiles), efficiency parameters, and animal health performance within a single study despite the critical nature of all these parameters in the sustainability of the dairy production system. Therefore, the aim of this study was to assess (at the herd level) (i) the impact of organic management on milk yield, basic composition, herd feed efficiency parameters, FA profiles, and animal health and (ii) the temporal variation of the observed differences throughout the year, along with (iii) the relative importance of the breeding and feeding drivers on efficiency, productivity, milk quality, and health parameters. The study hypothesized that organic management practices might reduce efficiency and productivity performance but will improve milk quality and cow health parameters, with animal diet and breed being significant drivers of the observed differences when compared with the conventional system.

## 2. Materials and Methods

### 2.1. Experimental Design and Collection of Data and Milk Samples

Milk samples were collected from 67 dairy herds in Southern England between January and December 2019, totaling 800 samples. A total of 26 farms were certified organic according to the Soil Association or Organic Farmers and Growers standards. Of these farms, 11 applied all-year-round calving, 9 applied autumn calving, 2 applied spring and autumn calving, and 4 applied spring calving. The remaining 41 farms were conventional, among which 1 farm applied spring and autumn calving, another applied spring calving, and the remaining 39 applied all-year-round calving. The differing calving systems may reflect individual farm goals that allow for block calving systems to utilize grassland during peak milk production, driving down production costs and all-year-round systems to produce consistent milk yields at the herd level all year round [13]. All farms practiced grazing between March and October. Perennial ryegrass dominated the pastures and silages for the conventional herds, while organic farms used pastures and silages of varying grass-to-clover ratios.

In order to enable an investigation into the seasonal variation of the measured parameters within each production system, the milk samples from bulk tanks containing milk (from more than one milking) were collected using a dipper from the top of the tank after agitating for at least 10 min to ensure that the milk was thoroughly mixed and the sample was representative on the third week of each month (between January and December 2019). Corresponding questionnaires, completed by each producer through an online questionnaire (where necessary, in cases that the data were not clear, a follow-up call by an interviewer), gathered at each sampling date, recorded information on breed (proportion of each breed or crossbreed in the herd based on farmers records) and feeding practices (type and amounts of conserved forage and other feeds and supplements offered). A total of 72 dairy farmers were invited to complete the questionnaire and provide milk samples, and eventually, 67 farms (93% of the invited) provided adequate data and milk samples to be included in the final database. Herd liveweight was estimated based on average breed liveweights and the contribution of breeds in the herd, as described by Qin et al. [14]. Offered DM when the cows were housed indoors included the summary of offered DM, as reported in farmers’ questionnaires. During the grazing season, in order to estimate pasture intake (not feasible to measure at commercial farms), total DMI was estimated using a prediction equation [4], and then pasture intake was estimated by difference (predicted DMI minus offered DM from concentrates and conserved forages, as reported in farmers’ questionnaires).

### 2.2. Milk Analysis

Milk samples were collected, kept at a refrigeration temperature, and were transferred within 24 h to the laboratory, where each sample was aliquoted into 2 different containers. One container (7 mL) was immediately frozen at −20 °C for further lyophilization (for subsequent FA profiling), while one chilled container (30 mL) was preserved with bronopol and sent to the National Milk Laboratories (Wolverhampton, UK) for fat, protein, and lactose analysis by Fourier transform infrared spectroscopy (FTIR) (MilkoScanTM 7RM; FOSS, Hillerød, Denmark) and somatic cell count (SCC) by flow cytometry (FossomaticTM 7; FOSS, Denmark).

Extraction, methylation, and esterification of lipids in the lyophilized milk were performed according to previously published methods [15], with the appropriate modifications in the following chromatographic conditions to achieve optimum peak separation. In brief, 130 mg of lyophilized milk was weighed into a glass tube and mixed with 2 mL hexane and 2 mL of 0.5 M sodium methylate and vortexed after each step. Tubes were placed at 50 °C in a dry block incubator (Thermo Scientific, Bellefonte, PA, USA) for 15 min and allowed to reach room temperature before adding 75 μL 12 N hydrochloric acid and were gently stirred. After leaving the tubes at room temperature for 15 min, 3 mL of hexane and 3 mL of deionized water were added and vortexed after each step. The tubes were centrifuged at 1160× *g* for 5 min at 5 °C, and the upper layer was collected for injection to a gas chromatograph (Agilent, 8890 GC system, Santa Clara, CA, USA) equipped with a flame ionization detector and a Varian CP-SIL 88 fused silica column (100 × 0.25 mm ID, 0.2 µm film thickness). At the injection port, the temperature was 255 °C, the pressure was 30psi, the septum purge flow was 3 mL/min, and a split ratio of 56:1 was used. At the FID port, the temperature was 260 °C, and the air, H2, and make-up (N2) flow was 300 mL/min, 30 mL/min, and 25 mL/min, respectively. Columns operated at a constant pressure of 30 psi. Oven temperature started at 70 °C, where it was kept for 4 min. It was then increased to 110 °C at a rate of 8 °C/min, and then (without hold time at 110 °C) to 170 °C at a rate of 5 °C/min, where it was kept for 14 min. Temperature was then increased to 240 °C at a rate of 3 °C/min and was kept for 12 min, resulting in a total runtime of 70.3 min. Peak identification relied on an external standard, including 52 FA methyl esters (GLC463, NU Check Prep, Elysian, MN, USA) and previously published milk chromatograms and methodologies [5]. A correction for carbon deficiency in the FID response for FA methyl esters with 4–10 atoms of carbon was used, as previously recommended [16]. An example chromatogram that illustrates the peak separation is presented in Figure S1 in the Supplementary Material. Human-health-related indices (AI; atherogenicity index, TI; thrombogenicity index, and HH ratio: hypocholesterolemic to hypercholesterolemic ratio) and desaturase activity index were calculated, as previously shown [17,18]. Methods to calculate FA intake of different population demographics based on milk FA profiles have been previously reported [18]. Sample collection and laboratory analyses were performed as blind, where the treatments were coded.

### 2.3. Statistical Analysis

A linear mixed model, repeated measures design analysis was carried out (residual maximum likelihood analysis; REML) using GenStat^®^ 18 [19] to investigate the effect of the production system, month, and their interaction on milk yield, basic composition, efficiency parameters, milk FA profiles, and health parameters. Farm was fitted as the subject and month as the time. The fixed effects were the production systems (conventional, organic), month (January–December) and their interaction, fitted in a factorial arrangement, while farm ID was included as a random effect. When the fixed effect was significant (*p* < 0.05), pairwise comparisons of the means were performed using Fisher’s least significant difference test. Residuals were assessed visually, and all variables were found to be normally distributed, except for lameness (% of herd), which was log(x + 1) transformed before analysis. Descriptive statistics to generate means and standard errors for presentation in tables were carried out in Minitab^®^ 20.2. In total, there were 10 missing milk samples (1.2% of the expected data): two in January, February, November, and December (3% of the within-month samples) and one in March and October (1.5% of the within-month samples). Multivariate redundancy analysis (RDA) was carried out using Canoco5^®^ [20] to further investigate the impact of production system and diet on productivity, the basic milk composition and efficiency parameters, the FA profiles, and the health parameters. Two RDA biplots were created: one for (i) productivity, basic composition, efficiency parameters, and health parameters and one for (ii) FA profiles. The arrow lengths and directions represent the correlations between the driver variables (diet composition parameters and breed) and response variables (basic composition, efficiency, health parameters, and FA profiles). All differences presented in the Results section (and subsequently discussed) are statistically significant (*p* < 0.05) unless otherwise stated. Exact *p*-values for all significant and nonsignificant differences in the measured variables between the production systems, months, and their interaction are presented in detail in Table 1, Table 2 and Table 3 in the main body of the present manuscript and Tables S2–S6 in the Supplementary Material.

## 3. Results

### 3.1. Herd Composition and Feed Intake

#### 3.1.1. Effect of Production System

Conventional and organic farms did not differ in their breed composition (Table 1), with the exception of Shorthorn (0.01–0.76%) and Ayrshire, which were higher in organic farms (+0.75% and +5.54% of the herd, respectively) and Guernsey, which were marginally higher for conventional farms (+0.06% of the herd). The Holstein breed was the most abundant breed in both the conventional and organic systems (80% and 66%, respectively). Offered DM was higher in conventional farms compared with organic farms (+2.1 kg DM/cow per day) (Table 1). The organic systems had a higher contribution of total forage (+13.2% offered DM), grazing (+16.6%), grass silage (+9.2%), grass:clover silage (+2.3%), cereal silage (+0.3%), other mixed silage (+2.1%), wholecrop silage (+7.8%), and cereals (+1.6%) when compared with the conventional systems. The conventional systems had significantly higher dietary contributions (% offered DM/cow per day) of total concentrates (+13.2 %), maize silage (+23.9 %), moist byproducts (+4.4 %), dry straights (+9.6 %), oil (+0.8 %), and minerals (63 g/cow/day) than in the organic herds. All other dietary ingredients did not differ between the two production systems. The ranges for herd and diet composition are displayed in Table S1 in the Supplementary Material.

**Table 1:** Means ± SE and *p*-values for the breed and diet composition of herds (%) from 67 farms, differing in production systems (conventional or organic) in Southern England.

	Production System				Production System × Month
	Conventional	Organic	SE	*p*-Value ^2^	*p*-Value ^2^
	*n* = 488 ^1^	*n* = 312 ^1^			
Milking herd size (number of cows)	270	211	5.2	0.045	<0.001
Milking cows (% herd)	86.3	84.9	0.50	0.638	<0.001
Estimated liveweight (kg) ^3^	656	637	2.4	0.111	0.181
Breed composition (% herd)					
Holstein	80.2	66.4	1.90	0.271	0.183
British Friesian	3.85	2.38	0.656	0.764	0.091
NZ Friesian	0.10	1.28	0.310	0.421	0.134
Jersey	0.35	0.81	0.110	0.476	0.313
Scandinavian Red	0.12	0.89	0.098	0.128	0.635
Shorthorn	0.01	0.76	0.035	0.001	0.018
Ayrshire	2.60	8.14	1.002	0.002	0.991
Montbeliarde	0.06	0.05	0.021	0.779	0.762
Brown Swiss	0.34	1.90	0.228	0.284	0.133
Guernsey	0.06	0.00	0.007	0.009	0.987
Other breed or crossbreed	12.3	17.4	1.68	0.534	0.356
Diet composition (% DM offered unless otherwise stated)					
Offered feed (kg DM/day) ^4^	21.0	18.9	0.11	<0.001	0.006
Total forage	61.0	74.2	0.46	<0.001	0.089
Total concentrate	39.0	25.8	0.46	<0.001	0.089
Predicted grazing intake	8.31	24.9	1.10	<0.001	<0.001
Total silage intake	50.2	47.7	0.97	0.720	<0.001
Grass silage	23.3	32.5	0.84	<0.001	<0.001
Grass:clover silage ^5^	0.86	3.19	0.329	0.018	<0.001
Maize silage	24.9	0.97	0.427	<0.001	0.056
Cereal silage	0.02	0.36	0.074	0.036	0.033
Lucerne silage	0.37	0.06	0.060	0.451	0.928
Other mixed silage	0.30	2.40	0.204	<0.001	0.014
Wholecrop	0.45	8.23	0.316	<0.001	<0.001
Hay and straw	2.46	1.95	0.166	0.723	0.010
Moist byproducts	4.74	0.37	0.203	<0.001	0.050
Dry straights ^6^	10.5	0.91	0.412	<0.001	0.922
Cereals	2.45	4.02	0.245	0.034	0.008
Blends	20.5	20.4	0.59	0.835	0.099
Oil	0.83	0.01	0.028	<0.001	0.947
Minerals (g/cow per day)	134.0	71.5	4.98	0.013	0.144
Vitamins (g/cow per day)	10.8	0.00	1.373	0.091	0.041

^1^ *n* is the number of records used to calculate means ± SE and *p*-values. Records with missing values were not included in the analysis. ^2^ Significances were declared at *p* < 0.05. ^3^ Average herd liveweights were estimated based on average breed liveweights and the proportionate number of cows from each breed or crossbreed in the total herd, as described by Stergiadis et al. [21]. ^4^ When cows had no access to pasture, this value reflects the summary of individual feed DM, as recorded in farmers’ questionnaires. During the grazing season, this is predicted using equations published by Butler et al. [4]. ^5^ In the conventional production system, this was predominantly perennial ryegrass silage, while in the organic systems, silage typically had variable grass:clover ratios. ^6^ Dry straights: single feedstuffs from which compound feeds and protein concentrates are prepared (wheat, barley, flaked maize, field beans, groundnut cake and meal, and soya bean meal).

#### 3.1.2. Effect of Month

No significant effects regarding month were found for breed composition. Offered DM varied between different months (highest in March and lowest in November), along with predicted grazing intake, total silage, grass silage, grass:clover silage, maize silage, lucerne silage, wholecrop silage, hay and straw, cereals, and vitamins. The results for herd and diet composition between January and December are displayed in Table S2 in the Supplementary Material.

#### 3.1.3. Effect of Production System × Month Interaction

There were monthly variations in the organic systems for the percentage of Shorthorn breeds within the herds, but no similar variation was detected for conventional systems, and no other interactions for production system and month were found regarding herd composition. There were significant production system × month interactions for most of the diet parameters (Table 1). Offered DM varied between the months in the organic and conventional systems. Predicted grazing was significantly higher in the organic systems between April and September compared to the conventional systems, with the largest difference being in May. The total proportion of silage was higher in the organic systems during the winter months but lower between April and July when compared with the conventional systems. Grass silage was highest in the organic systems from January to March and August to December but lower in May in the organic systems compared to the conventional systems. Grass:clover silage was higher in the conventional systems between January and March when compared with the organic systems. Cereal silage was higher in the conventional systems when compared to the organic systems in October and November. Other mixed silages contributed more to the total feed offered in November and December in the organic farms when compared to the conventional farms. Wholecrop silage was significantly higher in the organic systems between January and April and September and December when compared with the conventional systems. Hay and straw were higher in January and lower in March and June in the organic systems when compared with conventional systems. Cereal intake was higher in organic farms in March, October, and November when compared to the conventional farms.

### 3.2. Productivity, Efficiency, and Health Parameters

#### 3.2.1. Effect of Production System

The conventional production system resulted in higher yields (kg/cow per day) for milk (+7.3), energy-corrected milk yield (ECMY) (+7.5), fat (+0.27), and protein (+0.25) and saw higher milk concentrations (g/kg milk) of protein (+0.30), casein (+0.20 g), lactose (+0.40), and urea (+0.03) when compared with organic milk (Table 2). The conventional systems had higher efficiencies for feed (+0.23 kg milk/kg offered DM), milk fat (+8.6 g milk fat/kg offered DM), and milk protein (+8.1 g milk protein/kg offered DM) when compared with organic. However, the organic systems had higher non-grazing feed efficiency (+0.50 kg milk/kg non-grazing offered DM), feed concentrate efficiency (+1.23 kg milk/kg concentrate offered DM), fat non-grazing efficiency (+20 g milk fat/kg non grazing offered DM), fat concentrate efficiency (+51 g milk fat/kg concentrate offered DM), non-grazing protein efficiency (+17 g milk protein/kg non-grazing offered DM), and protein concentrate efficiency (+42 g milk protein/kg concentrate offered DM) when compared with conventional. The conventional systems resulted in more mastitis cases (+0.47 per 100 cows), a higher SCC (+14 × 10^3^/mL milk), and a lower fat-to-protein (F:P) ratio when compared with the organic systems (Table 2).

**Table 2:** Means ± SE and *p*-values for the effect of differing production systems on milk yield and basic composition, efficiency parameters, and health parameters from 67 farms across Southern England (41 conventional and 26 organic farms).

	Production System				Production System × Month
	Conventional	Organic	SE	*p*-Value ^2^	*p*-Value ^2^
	*n* = 488 ^1^	*n* = 312 ^1^			
Productivity (kg/cow per day)					
Milk yield	29.6	22.3	0.23	<0.001	<0.001
ECMY	30.3	22.8	0.22	<0.001	<0.001
Milk fat yield	1.15	0.88	0.008	<0.001	<0.001
Milk protein yield	0.99	0.74	0.007	<0.001	<0.001
Basic composition (g/kg milk)					
Milk fat	39.2	39.6	0.15	0.280	0.060
Milk protein	33.4	33.1	0.08	0.047	<0.001
Milk casein	26.3	26.1	0.08	0.048	<0.001
Milk whey protein	7.12	7.05	0.014	0.153	<0.001
Milk lactose (g/kg milk)	45.2	44.8	0.04	0.014	<0.001
Urea (g/kg milk)	0.17	0.14	0.003	<0.001	<0.001
Efficiency Parameters					
Feed efficiency (kg milk/kg DM offered)	1.41	1.18	0.011	<0.001	<0.001
Feed non-grazing efficiency (kg milk/kg non-grazing DM offered)	1.65	2.15	0.061	0.004	<0.001
Feed concentrate efficiency (kg milk/kg concentrate DM offered)	4.05	5.28	0.103	<0.001	0.001
Fat efficiency (g fat/kg DM offered)	55.2	46.6	0.410	<0.001	<0.001
Fat non-grazing efficiency (g fat/kg non-grazing DM offered)	64.7	84.8	2.42	0.004	<0.001
Fat concentrate efficiency (g fat/kg concentrate DM offered)	159	210	4.3	<0.001	0.002
Protein efficiency (g protein/kg DM offered)	47.1	39.0	0.333	<0.001	<0.001
Protein non-grazing efficiency (g protein/kg non-grazing DM offered)	55.3	72.3	2.13	0.007	<0.001
Protein concentrate efficiency (g protein/kg concentrate DM offered)	135	177	3.7	<0.001	<0.001
Health parameters and indicators					
Mastitis (% of herd)	2.63	2.16	0.090	0.031	0.310
Lameness (% of herd)	2.05	2.65	0.122	0.279	0.396
Other disease (% of herd)	0.75	0.63	0.060	0.262	0.033
Fat:protein	1.17	1.20	0.004	0.002	<0.001
Milk SCC (×1000/mL milk)	150	136	2.8	0.024	0.655

^1^*n* is the number of records used to calculate means ± SE and *p*-values. Records with missing values were not included in the analysis. ^2^ Significances were declared at *p* < 0.05.

#### 3.2.2. Effect of Month

All measured productivity, efficiency, and health parameters varied between the different months, except for lameness (% of herd). The effects of month on productivity, efficiency, and health parameters are displayed in Table S3 in the Supplementary Material.

#### 3.2.3. Effect of Production System × Month Interaction

**Figure 1:**
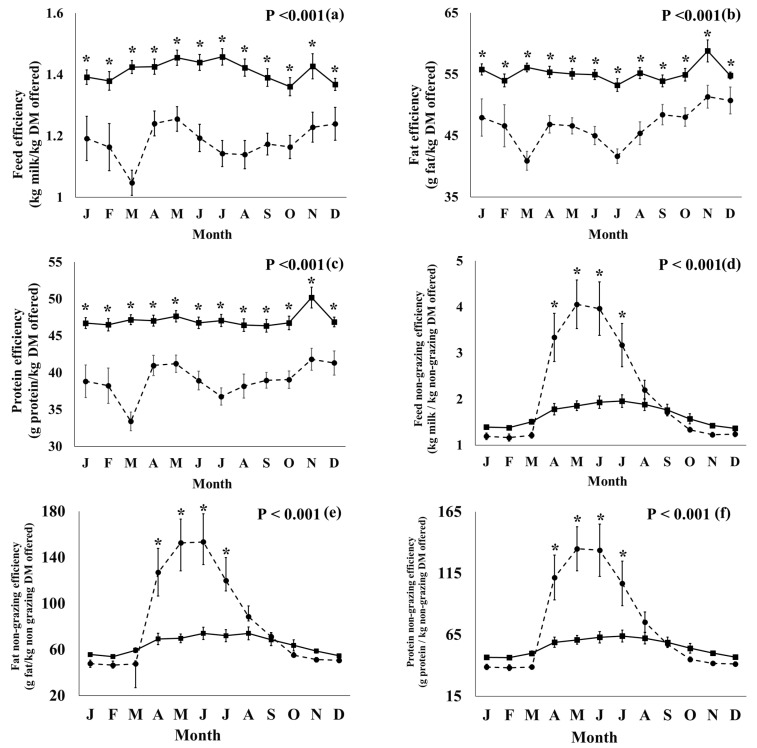

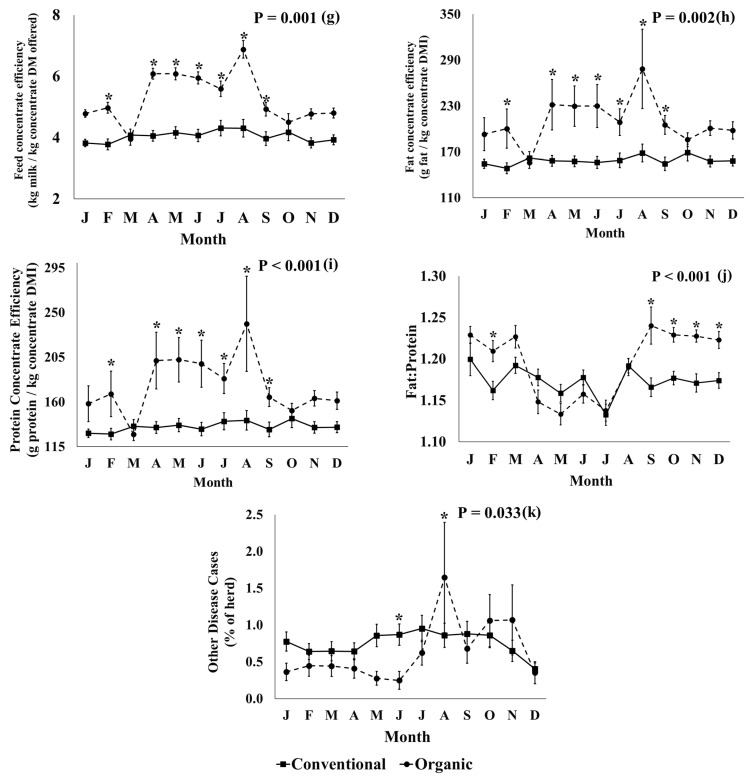
Feed efficiency and health parameters, where a significant (*p* < 0.05) interaction between production system (conventional, solid line; organic, dotted line) and month was observed. Units used: kg output/kg DM offered (panels (**a**,**d**,**g**)), g output/kg DM offered (panels (**b**,**c**,**e**,**f**,**h**,**i**)), fat:protein ratio of milk (**j**) and % of herd (**k**). Means were calculated from the measured values. *: significant difference between conventional and organic farms within the month (*p* < 0.05). The error bars represent the standard error of the means.

All productivity, efficiency, and health parameters showed significant production system × month interactions (Figure 1), except for SSC, mastitis, and lameness (% of herd). The conventional farms had more consistent feed, fat, and protein efficiency compared to the organic farms throughout the year, but the difference was smaller at the start of the grazing season (April–May). The organic systems had higher non-grazing feed efficiencies for feed, fat, and protein from April–July when compared with the conventional systems. The organic farms produced more milk, protein, and fat per kg of concentrate offered DM in February and between April and September when compared with the conventional farms. The organic farms had a lower percentage of other disease cases per herd in June but a higher percentage in August when compared with the conventional farms. The F:P ratio was higher in the organic milk in February and September when compared with the conventional milk.

#### 3.2.4. Multivariate Analysis of the Effect of Diet Composition on Milk Productivity, Efficiency, and Health Parameters

**Figure 2:**
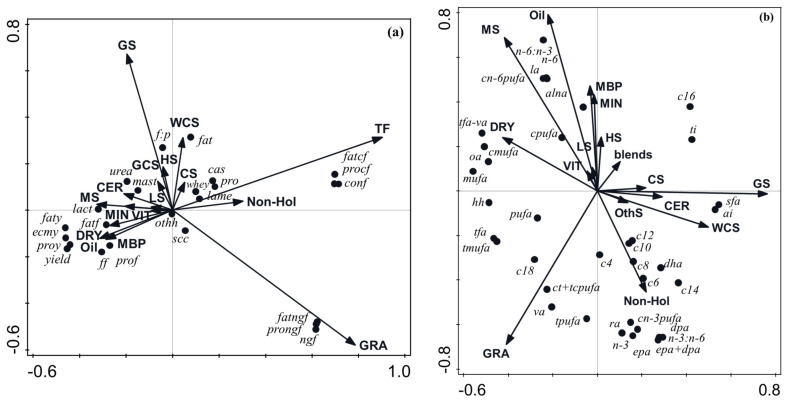
(**a**) Biplot derived from the redundancy analysis, showing the relationship between diet composition parameters (total forage, TF; estimated grazing, GRA; grass silage, GS; grass clover silage, GCS; maize silage, MS; lucerne silage, LS; cereal silage, CS; wholecrop silage, WCS; hay and straw, HS; moist byproducts, MBP; cereals, CER; Oil, Oil; dry straights, DRY; minerals, MIN; compound feed, COM), and non-Holstein genetics (Non-Hol) relative to (i) milk yield (kg/cow per day) *(yield)* and basic composition parameters, including milk fat yield (kg/cow per day) *(faty)*; milk protein yield (kg/cow per day) *(proy)*; milk fat content (g/kg milk) *(fat)*; milk protein content (g/kg milk) *(pro)*; energy-corrected milk yield *(ecmy)*; milk casein, g/kg milk *(cas)*; milk whey protein (g/kg milk) *(whey)*; milk urea content (g/kg milk) *(urea)*; milk lactose content (g/kg milk) *(lact)*; fat:protein ratio *(f:p)*; milk SCC (×1000/mL milk) *(scc)*; and (ii) efficiency parameters, including feed efficiency (kg milk/kg DMI) *(ff)*; feed non-grazing efficiency (kg milk/kg non-grazing DM offered) *(ngf)*; feed concentrate efficiency (kg milk/kg concentrate DM offered), *(conf)*; fat efficiency (g fat yield/kg DM offered) *(fatf)*; fat non-grazing efficiency (g fat yield/kg non-grazing DM offered) *(fatngf)*; fat concentrate efficiency (g fat yield/kg concentrate DM offered) *(fatcf)*; protein efficiency (g protein yield/kg DM offered) *(prof)*; protein non-grazing efficiency (g protein yield/kg DM offered) *(prongf)*; protein concentrate efficiency (g protein yield/kg concentrate DM offered) *(procf).* The total adjusted explained variation was 48.3%. Axis 1 explained 41.4% of variation, and Axis 2 explained a further 6.8% of the variation. Continuous variables, shown as arrows, were the following (presented in order of contribution to the explained variation; *p*-value also shown in parentheses): TF (34.4%, *p* = 0.002), GRA (10.3%, *p* = 0.002), MS (1.8%, *p* = 0.002), Oil (0.81%, *p* = 0.002), Non-Hol (0.71%, *p* = 0.002), HS (0.29%, *P* = 0.03), CER (0.29%, *p* = 0.016), GS (0.23%, *p* = 0.038), WCS (0.15%, *p* = 0.084), MBP (0.095%, *p* = 0.238), LS (0.67%, *p* = 0.252), MIN (0.05%, *p* = 0.418), GCS (0.03%, *p* = 0.542), CS (0.03%, *p* = 0.416), DRY (0.01%, *p* = 0.854), VIT (0.01%, *p* = 0.878). (**b**) Biplot derived from the redundancy analysis, showing the relationship between diet composition parameters (total forage, TF; estimated grazing, GRA; grass silage, GS; maize silage, MS; lucerne silage, LS; cereal silage, CS; wholecrop silage, WCS; hay and straw, HS; moist byproducts, MBP; cereals, CER; Oil, Oil; dry straights, DRY; minerals, MIN; compound feed, COM), and non-Holstein genetics (Non-Hol) relative to milk concentrations of butyric acid (*c4*), caproic acid (*c6*), caprylic acid (*c8*), capric acid (*c10*), lauric acid (*c12*), myristic acid (*c14*), palmitic acid (*c16*), stearic acid (*c18*), vaccenic acid (*va*), oleic acid (*oa*), linoleic acid (*la*), rumenic acid (*ra*), α-linolenic acid (*alna*), eicosapentaenoic acid (*epa*), docosapentaenoic acid (*dpa*), docosahexaenoic acid (*dha*), eicosapentaenoic acid + docosahexaenoic acid (*epa+dha),* saturated fatty acids (*sfa*), monounsaturated fatty acids (*mufa*), *cis*-monounsaturated fatty acids (*cmufa*), polyunsaturated fatty acids (*pufa*), *cis*-polyunsaturated fatty acids (*cpufa*), omega-3 fatty acids (*n3*), omega-6 fatty acids (*n6*), omega-3:omega-6 (*n3:n6*), *cis* n-3-polyunsaturated fatty acids (*cn-3pufa*), *cis* n-6 polyunsaturated fatty acids (*cn-6pufa*), omega-6:omega-3 (*n6:n3*), *trans* fatty acids (*tfa*), *trans* fatty acids excluding vaccenic acid (*tfa-va*), *cis,trans + trans,cis* polyunsaturated fatty acid (*ct + tcpufa*), atherogenicity index (*ai*), thrombogenicity index (*ti*), and hypocholesterolaemic to hypercholesterolaemic ratio (*hh*). The total adjusted explained variation was 32.4%. Axis 1 explained 23.6% of the variation, and Axis 2 explained a further 8% of the variation. Continuous variables, shown as arrows were the following (presented in order of contribution to the explained variation; *p*-value also shown in parentheses): GS (13.8%, *p* = 0.002), Oil (5.52%, *p* = 0.002), WCS (4.19%, *p* = 0.002), GRA (2.36%, *p* = 0.002), DRY (1.84%, *p* = 0.002), Blends (1.44%, *p* = 0.002), Non-Hol (1.49%, *p* = 0.002), MIN (0.65%, *p* = 0.004), MS (0.66%, *p* = 0.006), LS (0.40%, *p* = 0.016), MBP (0.41%, *p* = 0.012), VIT (0.31%, *p* = 0.038), CS (0.23%, *p* = 0.088), CER (0.17%, *p* = 0.116), HS (0.13%, *p* = 0.192), OthS (0.08%, *p* = 0.342).

An RDA biplot showing the impact of feed and breed drivers on productivity, efficiency, and health parameters is demonstrated in Figure 2a. The drivers, when taken together, explained 48.3% of the variation, of which 41.4% was explained by axis 1 and a further 6.8% was explained by axis 2. Total forage, grazing, and maize silage intake explained 34.4%, 10.3%, and 1.8%, respectively, while the remaining drivers explained <1% of the variation each. The intakes of total forage were positively associated with feed, fat, and protein concentrate efficiency and, to a lesser extent, cases of casein, protein, whey, and lameness. Grazing was strongly positively associated with feed, fat, and protein non-grazing efficiency and, to a lesser extent, SCC; it was negatively correlated with milk urea, F:P, and mastitis cases. Moist byproducts, oils, dry straights, vitamins, minerals, maize silage, lucerne silage, and cereals were negatively correlated with the efficiency parameters that total forage was a positive driver for, as well as milk protein, casein, whey content, and lameness cases, but was positively associated with (i) milk, protein and fat yield, ECMY, lactose, and (ii) milk, protein, and fat efficiency, which were the parameters that total forage intake was negatively correlated with. Grass silage, wholecrop silage, hay and straw, grass:clover silage, and, to a lesser extent, cereal silage were all positively associated with milk fat and urea content, F:P, and mastitis cases. The non-Holstein breeds and cereal silage were positively associated with milk casein, protein, and whey concentrations, as well as lameness cases; they were also positively correlated with concentrate use efficiencies but were negatively correlated with outputs of milk, fat, and protein.

### 3.3. Milk Fatty Acid Profile

#### 3.3.1. Effect of Production System

The organic milk had higher concentrations of (g/kg total FA) total SFA (+14) and individual SFA; C4:0 (+0.6), C6:0 (+0.8), C8:0 (+0.4), C14:0 (+7.0), individual MUFA; VA (+4.9), overall PUFA (+2.4) and individual PUFA, rumenic acid (RA, +2.1), ALNA (+2.4), eicosapentaenoic acid (EPA, +0.2), docosapentaenoic acid (DPA, +0.3), docosahexaenoic acid (DHA, +0.02), EPA+DHA (+0.3), *trans* PUFA (+0.2), *cis:trans + trans:cis* PUFA (+2.4), *cis* omega-3 PUFA (*cis* n-3 PUFA; +3.1), omega-3 (n-3, +4.2), omega-3:omega-6 (n-3:n-6, +0.3), and AI (+0.2) when compared with conventional milk (Table 3). The conventional milk had higher concentrations (g/kg total FA) of overall MUFA (+16), *cis* MUFA (+16), individual MUFA, oleic acid (OA, +13) and individual PUFA, linoleic acid (LA, +3.1), n-6 (+3.6), *cis* n-6 PUFA (+3.4), *trans* FA excluding VA (+4.7), n-6:n-3 (+1.29), and HH (+0.03). All the desaturase activity indicators were higher for the conventional milk, except for C14:1:C14:0, which was higher in the organic milk. The effect of the production system on all the individual FAs measured is displayed in Table S4 in the Supplementary Material.

**Table 3:** Means ± SE and *p*-values for the effect of production system on the milk fatty acid profile from 67 farms across Southern England with differing production systems (41 conventional and 26 organic farms).

	Production System				Production System × Month
	Conventional *n* = 485 ^1^	Organic *n* = 309 ^1^	SE	*p*-Value ^2^	*p*-Value ^2^
Individual FA (g/kg total FA)					
SFA					
C4:0	26.2	26.8	0.07	<0.001	<0.001
C6:0	18.4	19.2	0.06	<0.001	<0.001
C8:0	11.6	12.0	0.05	0.029	<0.001
C10:0	27.1	27.9	0.17	0.292	<0.001
C12:0	34.0	34.5	0.24	0.968	<0.001
C14:0	106	113	0.4	<0.001	0.122
C16:0	314	309	0.1	0.905	<0.001
C18:0	99.3	102	0.6	0.080	<0.001
MUFA					
VA (C18:1 *t*11)	11.9	16.8	0.28	<0.001	<0.001
OA (C18:1 *c*9)	198	185	0.89	<0.001	0.015
PUFA					
LA (18:2 *c*9*c*12)	19.1	16.0	0.21	<0.001	0.365
RA	5.94	8.00	0.123	<0.001	<0.001
ALNA (18:3 *c*9*c*12*c*15)	4.53	6.90	0.063	<0.001	0.013
EPA (20:5 *c*5*c*8*c*11*c*14*c*17)	0.44	0.66	0.006	<0.001	<0.001
DPA (C22:5 *c*7*c*10*c*13*c*16*c*19)	0.74	1.05	0.007	<0.001	0.002
DHA (C22:6 *c*4*c*7*c*10*c*13*c*16*c*19)	0.05	0.07	0.001	<0.001	0.002
FA groups (g/kg total FA)					
SFA ^3^	678	692	1.4	<0.001	<0.001
MUFA ^4^	279	263	1.2	<0.001	<0.001
*cis* MUFA ^6^	242	226	0.9	<0.001	0.018
*trans* MUFA ^7^	36.7	37.0	0.41	0.405	<0.001
PUFA ^5^	43.3	45.7	0.30	0.014	<0.001
*cis* PUFA ^8^	27.8	27.6	0.22	0.132	0.036
*trans* PUFA ^9^	0.24	0.41	0.009	<0.001	<0.001
*cis,trans + trans,cis* PUFA ^10^	15.3	17.7	0.19	<0.001	<0.001
n-3 ^11^	8.30	12.5	0.19	<0.001	<0.001
n-6 ^12^	22.7	19.1	0.234	<0.001	0.207
*cis* n-3 PUFA ^13^	5.91	8.97	0.073	<0.001	0.015
*cis* n-6 PUFA ^14^	21.8	18.4	0.23	<0.001	0.216
n-3:n-6 ratio	0.40	0.68	0.106	<0.001	<0.001
n-6:n-3 ratio	2.91	1.62	0.384	<0.001	<0.001
EPA+DHA	0.48	0.74	0.007	<0.001	<0.001
*trans* FA ^15^	38.5	38.6	0.44	0.526	<0.001
*trans* FA (exc. VA)	26.7	22.0	0.23	<0.001	0.028
Human health related indices					
AI ^16^	2.43	2.63	0.020	<0.001	<0.001
TI ^17^	2.92	2.88	0.019	0.529	<0.001
HH ^18^	0.54	0.51	0.004	0.004	0.011
Δ9-desaturase activity indicators					
Δ9I ^19^	0.28	0.27	0.001	<0.001	0.007
C14:1:C14:0	0.02	0.02	0.000	<0.001	<0.001
C16:1:C16:0	0.03	0.03	0.000	<0.001	0.009
OA:C18:0	2.01	1.81	0.008	<0.001	0.006
RA:VA	0.17	0.16	0.006	<0.001	<0.001

^1^ *n* is the number of records used to calculate means. ^2^ Significances were declared at *p* < 0.05. ^3^ SFA: C4:0, C5:0, C6:0, C7:0, C8:0, C9:0, C10:0, C11:0, C12:0, C13:0, C14:0, C15:0, C16:0, C17:0, C18:0, C20:0, C22:0, C24:0. ^4^ MUFA: C10:1 *c*9, C11:1 *c*10, C12:1 *c*9, C13:1, C14:1 *t*9, C15:1 *c*9, C16:1 *t*6-8, C16:1 *t*9, C16:1 *t*11 + *t*12 + *t*13, C16:1 (co-elutes with C17:0 anteiso, C16:1 *c*11, C16:1 *c*13, C17:1 *t*10, C17:1 *c*9, C18:1 *t*4, C18:1 *t*5, C18:1 *t*6-8, C18:1 *t*9, C18:1 *t*10, C18:1 *t*11 (VA), C18:1 *c*6 + *t*12, C18:1 *c*9 (OA), C18:1 t15, C18:1 *c*11, C18:1 *c*12, C18:1 *c*13, C18:1 *c*14 (co-elutes with C18:1 *t*6), C18:1 *c*15 (co-elutes with C19:0), C18:1 *c*16, C19:1, C20:1 *c*8, C20:1 *c*11, C22:1 *c*13, C24:1 *c*15. ^5^ PUFA: C18:2 *t*11*t*15, C18:2 *t*9*t*12, C18:2 *c*9*t*13, C18:2 *c*10*t*14, C18:2 *c*9*t*14, C18:2 *c*9*t*12, C18:2 *t*11*c*15, C18:2 *t*9*c*12, C18:2 *c*9*c*12 (LA), C18:2 *t*12*c*15, C18:3 *c*6*c*9*c*12, C18:3 *c*9*c*12*c*15 (ALNA), C18:2 *c*9*t*11 conjugated (RA) (co-elutes with C18:2 *t*7*c*9 + *t*8*c*10 + *t*6*c*8), other conjugated FA of unknown isomerisms of C18:2, C20:2 *c*11*c*14, C20:3 *c*8*c*11*c*14, C20:3 *c*11*c*14*c*17, C20:4 *c*5*c*8*c*11*c*14, C22:2 *c*13*c*16, C20:5 *c*5*c*8*c*11*c*14*c*17 (EPA), C22:3 *c*13*c*16*c*19, C22:4 *c*7*c*10*c*16*c*19, C22:5 *c*7*c*10*c*13*c*16*c*19 (DPA), C22:6 *c*4*c*7*c*10*c*13*c*16*c*19 (DHA). ^6^ *cis* MUFA: C10:1 *c*9, C11:1 *c*10, C12:1 *c*9, C13:1 *c*9, C14:1 *c*9, C16:2 *c*9 (co-elutes with C17:0 anteiso), C16:1 *c*11, C16:1 *c*13, C17:1 *c*9, C18:1 *c*6 (co-elutes with C18:1 *t*16), C18:1 *c*9 (OA), C18:1 *c*11, C18:1 *c*12, C18:1 *c*13, C18:1 *c*14 (co-elutes with C18:1 *t*6), C18:1 *c*15 (co-elutes with C19:0), C18:1 *c*16, *c*19:1 *c*9, C20:1 *c*8, C20:1 *c*11, C22:1 *c*13, C24:1 *c*15. ^7^ *trans* MUFA:C14:1 *t*9, C16:1 *t*6 + *t*7 + *t*8, C16:1 *t*9, C16:1 *t*11 + *t*12 + *t*13, C17:1 *t*10, C18:1 *t*4, C18:1 *t*5, C18:1 *t*6+*t*7+*t*8, C18:1 *t*9, C18:1 *t*10, C18:1 *t*11 (VA), C18:1 *t*12 (co-elutes with C18:1 *c*6), C18:1 *t*15, C18:1 *t*16 (co-elutes with C18:1 *c*14). ^8^ *cis* PUFA:C18:2 *c*9*c*12 (LA), C18:3 *c*6*c*9*c*12, C18:3 *c*9*c*12*c*15 (ALNA), 20:2 *c*11*c*14, C20:3 *c*8*c*11*c*14, C20:3 *c*11*c*14*c*17, C20:4 *c*5*c*8*c*11*c*14, C22:3 *c*13*c*16, C20:5 *c*5*c*8*c*11*c*14*c*17 (EPA), C22:3 *c*13*c*16*c*19, C22:4 *c*7*c*10*c*13*c*16, C22:5 *c*7*c*10*c*13*c*16*c*19 (DPA), C22:6 *c*4*c*7*c*10*c*13*c*16*c*19 (DHA). ^9^ *trans* PUFA: C18:2 *t*11*t*15, C18:2 *t*9*t*12. ^10^ *cis,trans* + *trans,cis* PUFA: C18:2 *c*9*t*13, C18:2 *c*10*t*14, C18:2 *c*9*t*14, C18:2 *c*9*t*12, C18:2 *t*11*c*15, C18:2 *t*9*c*12, C18:2 *t*12*c*15, C18:2 *c*9*t*11 (RA) (co-elutes with C18:2 *t*7*c*9 + *t*8*c*10 + *t*6*c*8), other conjugated FA of unknown isomerism (CLA other a-h). ^11^ omega-3 PUFA (n-3): C18:2 *t*11*t*15, C18:2 *t*11*c*15, C18:2 *t*12*c*15, C18:3 *c*9*c*12*c*15, C20:3 *c*11*c*14*c*17, C20:5 *c*5*c*8*c*11*c*14*c*17 (EPA), C22:3 *c*13*c*16*c*19, C22:5 *c*7*c*10*c*13*c*16*c*19 (DPA), C22:6 *c*4*c*7*c*10*c*13*c*16*c*19. ^12^ omega-6 PUFA(n-6): C18:2 *t*9*t*12, C18:2 *c*9*t*12, C18:2 *t*9*c*12, C18:2 *c*9*c*12 (LA), C18:3 *c*6*c*9*c*12, C20:2 *c*11*c*14, C20:3 *c*8*c*11*c*14, C20:4 *c*5*c*8*c*11*c*14, C22:2 *c*13*c*16, C22:4 *c*7*c*10*c*13*c*16. ^13^ *cis* n-3 PUFA: C18:3 *c*9*c*12*c*15, C20:3 *c*11 *c*14 *c*17, C20:5 *c*5*c*8*c*11*c*14*c*17, C22:3 *c*13*c*16*c*19, C22:5 *c*7*c*10*c*13*c*16*c*19, C22:6 *c*4*c7c*10*c*13*c*16*c*19. ^14^ *cis* n-6 PUFA: C18:2 *c*9*c*12, C18:3 *c*6*c*9*c*12, C20:2 *c*11*c*14, C20:4 *c*5*c*8*c*11*c*14, C22:2 *c*13*c*16. ^15^ *trans* FA: C14:1 *t*9, C16:1 *t*6+*t*7+*t*8, C16:1 *t*9, C16:1 *t*11 + *t*12 + *t*13, C17:1 *t*10, C18:1 *t*4, C18:1 *t*5, C18:1 *t*6 + *t*7 + *t*8, C18:1 *t*9, C18:1 *t*10, C18:1 *t*11 (VA), C18:1 *t*12 (co-elutes with C18:1 *c*6), C18:1 *t*15, C18:1 *t*16 (co-elutes with C18:1 *c*14), C18:2 *t*11*t*15, C18:2 *t*9*t*12. ^16^ Atherogenicity index = (C12:0 + (4 × C14:0) + C16:0)/(MUFA + PUFA), as described in Średnicka-Tober*,* et al. [22]. ^17^ Thrombogenicity index= (C14:0 + C16:0 + C18:0)/(0.5 × MUFA) + (0.5 × n-6) + (3 × n-3) + (n-3:n-6) as described in Średnicka-Tober, et al. [22]. ^18^ Hypocholesterolemic to hypercholesterolemic ratio = (C18:1 *c*9 + total PUFA)/(C12:0 + C14:0 + C16:0) as described in Mierlita [17]. ^19^ Δ9-desaturase activity index = (*c*9 C14:1 + *c*9 C16:1 + OA + RA)/(*c*9 C14:1 + *c*9 C16:1 + OA + RA + C14:0 + C16:0 + C18:0 + VA), as described in Kay et al. [23].

#### 3.3.2. Effect of Month

All the measured FAs displayed in Table 3 showed variations between months and are outlined in Table S5 in the Supplementary Material. All the FA concentrations across the different months are displayed in Table S6 of the Supplementary Material.

#### 3.3.3. Effect of Production System × Month Interaction

**Figure 3:**
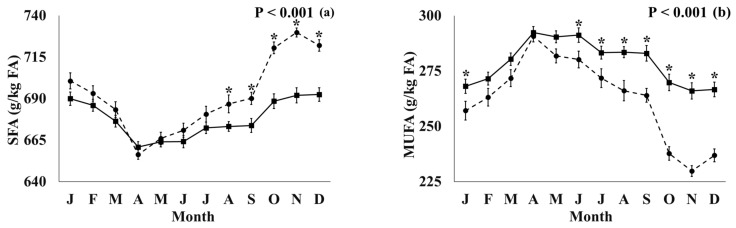

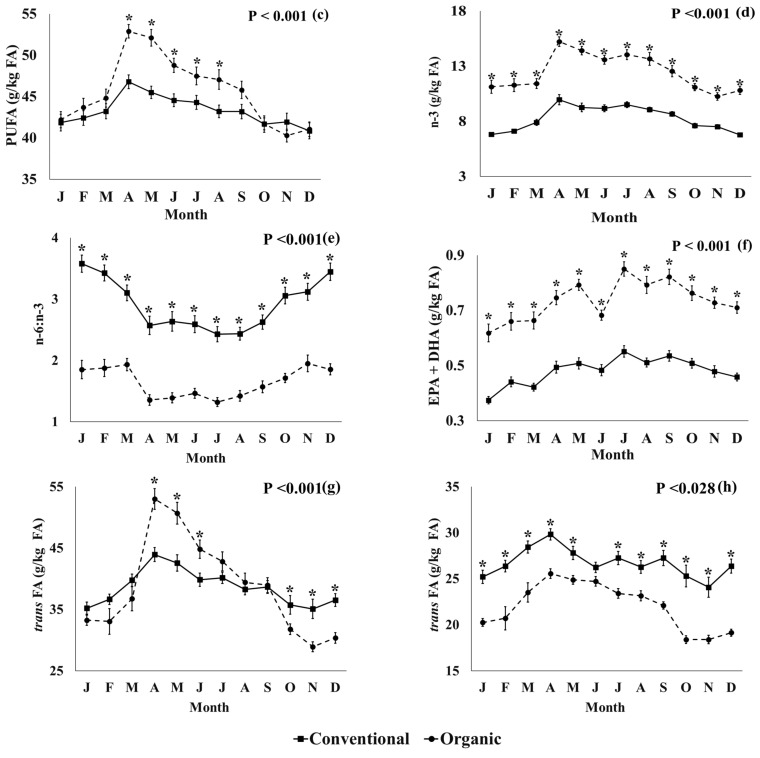
Fatty acid groups ((**a**) SFA; (**b**) MUFA; (**c**) PUFA; (**d**) n-3; (**e**) n6:n3; (**f**) EPA + DHA; (**g**) trans FA; (**h**) trans FA (no VA)), where a significant (*p* < 0.05) interaction between the production system (conventional, solid line; organic, dotted line) and the month was observed. *: significant difference between conventional and organic farms within the month (*p* < 0.05). Means were calculated from the measured values. The error bars represent the standard error of the means.

The significant interactions between production system *×* month were found for all FA displayed in Table 3, except for C14:0, LA, n-6, and *cis* n-6 PUFA. The significant interactions for the FA groups, which are associated with specific reference nutrient intakes in human diets, are shown in Figure 3. Significant interactions for individual FA and FA groups are displayed in Figures S2–S4 in the Supplementary Material. Organic milk had significantly higher concentrations of SFA between August and December, with the largest difference being in November (+37.9 g/kg FA), when compared to the conventional milk. The opposite was found for MUFA, with organic milk being significantly lower in MUFA than conventional milk between June and December, with the largest difference appearing in November (−36.2 g/kg FA). The organic milk had higher PUFA concentrations between April and August, with the largest difference being in May (+6.6 g/kg FA). Omega 3 and EPA + DPA concentrations were higher in the milk from the organic farms across all months, with the largest differences occurring in April (+0.54 g/kg FA) and July (+0.30 g/kg FA), respectively, but the opposite was found for n-6:n-3, with the largest difference appearing in January. *Trans* FA was higher in the organic milk between April and July, with the largest difference appearing in April (+9.06 g/kg FA) and lower differences between October and December. *Trans* FA (no VA) was higher in the conventional milk across all months, with the largest difference appearing in October (+6.91 g/kg FA).

#### 3.3.4. Multivariate Analysis of the Effect of Diet Composition on Milk Fatty Acid Profile

The RDA biplot showing the impact of feed and breed drivers on the FA profile is demonstrated in Figure 2b. The drivers, when taken together, explained 32.4% of the variation, of which 23.6% was explained by axis 1, and a further 8.0% was explained by axis 2. Grass silage, oil, wholecrop silage, grazing, dry straights, blends, and non-Holstein explained 13.8, 5.53, 4.19, 2.36, 1.84, 1.44, and 1.49%, respectively, while the remainder explained <1% of the variation of each. Intakes of grass silage, wholecrop silage, cereals, cereal silage, and other silages were positively associated with total SFA and all individual SFAs from C6:0–C16:0, AI, and TI and negatively correlated with MUFA (total, *cis* and *trans*), *trans* FA (total and excluding VA), C18:0, OA, and PUFA. Non-Holstein breeds and grazing were positively associated with individual SFAs from C4:0–C14:0, DHA, DPA, EPA, EPA+DHA, RA, n-3, *cis* n-3 PUFA, n-3:n-6, *trans* PUFA, *cis:trans + trans:cis* PUFA, VA, C18:0, *trans* FA, *trans* MUFA, PUFA, and HH. Grazing was negatively correlated with C16:0 and TI, while non-Holstein breeds were negatively correlated with LA, n-6, n-6:n-3, *cis* n-6 PUFA, *cis*PUFA, and ALNA. Dry straights, maize silage, oil, minerals, moist byproducts, and, to a lesser extent, vitamins and lucerne silage, were positively associated with MUFA, OA, *cis* MUFA, *trans* FA without VA, *cis* PUFA, *cis* n-6 PUFA, LA, n-6:n-3, n-6, and ALNA and negatively correlated with the FA groups that were positively associated with the non-Holstein breeds.

## 4. Discussion

### 4.1. Effect of Production System on Productivity and Efficiency

Milk yield and ECMY in the conventional herds were higher than in the organic ones, as was observed in previous studies [5,11,24], probably as a result of increased concentrate intake [11] and the influence of Holstein genetics that are associated with high-intensity and conventional systems [5]. This was supported by RDA, showing positive associations between yield and intakes of concentrate ingredients and negative associations with non-Holstein genetics.

Previous studies have reported higher concentrations of fat, protein, and casein in organic or low-input farms when compared to conventional or high-intensity farms and have attributed this to the potential influence of non-Holstein breeds, which are often utilized in low-input and organic systems [5]. However, the current study found no significant differences in non-Holstein breeds and milk fat between the two production systems. Although the protein and casein content was higher in the conventional milk [7,25], probably due to the higher inclusion of concentrates in conventional diets [26]. Interestingly, the milk protein concentrations were higher from the conventional farms in January, March, October, and November but were higher in the organic milk in August. The RDA indicates a positive correlation between milk protein and intake of total forage, which was higher in the organic farms throughout the year and highest in August, contributing +15.9% to offered DM when compared to the conventional farms, and this could explain higher protein content in the organic milk in August.

When considering the significant reliance on fresh herbage and conserved forages in organic systems, the efficient conversion of feed, particularly that of conserved forages and grazing, to milk is essential [8]. In line with previous investigations [11,12,27], the current study found that conventional herds were more efficient converters of feed to milk, fat, and protein. Although measures of efficiency differed between the studies: Brito and Silva [12], Gaudaré et al. [11], and Lorenz et al. [27] all reported efficiency with regard to ECMY per kg DMI, kg milk per kg DMI, and FPCM per kg DMI, respectively. In our study, we used the kg milk per kg offered DM as an indicator of efficiency. However, the results were similar even when we used ECMY as the measured output (instead of milk yield) in the calculation of efficiency parameters. In line with Gaudaré et al. [11], the current study found organic herds produced more milk, fat, and protein per kg of non-grazing and concentrate offered as a result of the lower inclusion of concentrate feeds in the organic diets, with these herds consuming, on average, 13.2% less than conventional herds, and the higher contribution of grazing in the diet (24.9% in organic vs. 8.30% in conventional). However, this improved efficiency in the organic systems was only observed when grazing was highest, and other dietary ingredients (conserved forage and concentrates) were lowest (between April and July). The results are supported by RDA, suggesting that the higher inclusion of concentrate feeds was negatively associated with non-grazing and concentrate feed efficiency. Concentrate and non-grazing feed efficiency should be taken into consideration, particularly in organic and pasture-based systems, due to the potential implications to farm profitability (concentrates being the most expensive part of the diet), as well as sustainability [11]. Findings of higher non-grazing and concentrate efficiency in organic farms support the utilization of grazing, particularly in organic or low-input systems during summer months in reducing the requirement for energy-rich, food-competing concentrate feeds [11].

However, it should be noted that offered feed DM in the present study, which is used in the calculation of feed efficiency, is not an actual measurement but depends either on farmer records (collected via questionnaires) during the time of the year that the cows had no access to pasture, or has been predicted based on average breed body weight and milk yield, as previously shown [4], during the grazing season because measuring pasture intake in commercial grazing herds is not feasible. Although, the supply of conserved forage and concentrate feeds at the whole-herd level (which represented a substantial amount of the offered diet of 92% and 75% DM for conventional and organic, respectively, across the survey) and at the herd level did not rely on predicted values and has been recorded via questionnaires. This approach may include more error in the estimates of the efficiency parameters when compared with collecting data from individual animals in a research environment. However, the results around feed efficiency are in line with previous work in pasture-based animals, which appears to confirm that, while overall feed efficiency might be superior in conventional systems [5,11,24], when concentrate feeding is common practice, increased grazing reduces the reliance on expensive concentrates and non-grazing ingredients [11].

In addition, when comparing the use efficiencies of diet ingredients between production systems with different ingredient inclusion rates, these should be interpreted with caution. The higher non-grazing and concentrate efficiencies found in the organic production systems revealed the importance of pasture inclusion in the diets of cows, with a simultaneous reduction in food-competing expensive ingredients, which highlights the opportunities to improve within-system profitability and sustainability by increasing pasture intake. The current data cannot be used to justify any potential increase in concentrate or nonpasture feeds in organic cows’ diets, as they do not show that organic herds will use the same amount of concentrate or nonpasture ingredients more efficiently than a conventional herd.

### 4.2. Effect of Production System on Animal Health

Results from the current study report that organic herds had fewer mastitis cases, expressed as a % of the herd, than the conventional herds, which is in agreement with Ellis et al. [28]. On the contrary, Stergiadis et al. [5] found no difference in mastitis cases between herds from different production intensities (spanning from organic to highly intensive), although, numerically, the higher intensity systems had more cases of mastitis, despite the use of preventative antibiotics. However, the RDA in the current study, which also agrees with Stergiadis et al. [5], identified negative associations between grazing and mastitis cases. Therefore, the high pasture intakes in organic systems are probably less relevant to mastitis. Ellis et al. [28] and Ward et al. [29] mentioned that farms with superior cow cleanliness had lower mastitis cases and SCC, but organic and conventional systems did not differ in cow cleanliness during the outdoor grazing period in their study [28]. Cleaning and milking strategies have not been recorded in this study, and it is not possible to comment in relation to the potential impact on mastitis cases. However, previous studies associated genetic selection for a high milk yield (typical in the Holstein breed, which was used more extensively in the conventional herds in the present study) and SCC and, subsequently, mastitis [30]. The organic herds in the current study had lower milk yields and SCC, as well as a higher contribution of lower-yielding breeds (Ayrshire and Shorthorn), which may have contributed to the lower cases of mastitis.

The F:P ratio has been used as an indicator of energy balance and is usually elevated in response to negative energy balance [31]. Additionally, studies have identified milk F:P as a suitable indicator for ketosis (F:P > 1.5) and acidosis risk (F:P < 1.0) [32]. Despite the differences in the F:P ratio between the organic and conventional herds, they were both within the normal limits of 1.1–1.5 [33] throughout the year, and this difference cannot be associated with any risks to animal health. The lower F:P found for conventional herds, although being within normal limits [33], could be a result of lower ruminal pH, which is associated with a higher intake of concentrate feeds, following the digestion of sugars by propionate-producing fermentation pathways, also known to produce lactate [34]. The ratio of F:P was higher in organic herds in February and between September and December and reflected the seasonal variation of the dietary contributors, with higher grazing and lower concentrate contribution between March and September, during which F:P was lowest. Studies have reported increases in the protein content of milk with increases in fresh herbage intake and a decrease in the fat content of milk in grazing cattle [35], which supports the finding of lower F:P during the grazing season in organic milk. The temporal variation in F:P in conventional milk was not as extensive due to the relatively stable forage-to-concentrate ratios in the conventional diets across the year.

### 4.3. Effect of Production System on Milk Fatty Acid Profile

Previous investigations have found lower SFA concentrations in low-input and organic milk when compared with high-intensity and conventional milk [4,5]. However, the current study reports the opposite. In previous studies, there was lower SFA in organic milk when compared with highly intensive herds [9], and this has been attributed to the increased fresh herbage intake associated with these systems, although differences were not observed between the organic and conventional systems, where the difference in pasture intake was 123 g/kg DM [9]. This may also be the case in the present study, as the difference in the contribution of total forage and pasture in the conventional and organic herds was less than 131 g/kg and 166 g/kg DM per day, respectively. The results from the RDA suggested that intakes of wholecrop and grass silage are stronger drivers for milk SFA than grazing. This is supported by Ellis et al. [3], who found that feeding with wholecrop and grass silage increased milk SFA, and Ormston et al. [36] found SFA to be positively correlated with intakes of grass and maize silages. When considering that organic diets had, on average, +778 g/kg DM and 920 g/kg DM more wholecrop and grass silage, respectively, than conventional diets, this may have further contributed to the higher SFA in organic milk in the current study. There is also abundant evidence identifying breed as a significant influencer of FA profile, observing lower milk SFA concentrations in the Holstein-Friesian cows when compared with alternative breeds [36,37]. The results from RDA in the current study also illustrated a positive correlation between some individual FAs (C6:0, C8:0, C10:0, C12:0, and C14:0) and non-Holstein breeds. Although, the differences between the systems for the breeds were not statistically significant (conventional herds had approximately 14 more Holstein cows) than the crossbreeds per 100 cows when compared with organic herds. This numerical difference in herd composition may have contributed to an increase in the individual milk SFA, such as C6:0, C8:0, and C14:0. Notably, the SFA in organic milk was higher only from September–December when pasture intake is minimal or zero in both systems. This is also in agreement with Butler et al. [4], who reported an increase in milk SFA from organic farms when compared to nonorganic low-input farms during the indoor period in August and October.

The results found in the current study for MUFA concentrations agree with the findings of Ellis et al. [3], where organic herds had a lower overall proportion of MUFA compared with conventional herds. However, other previous investigations report higher proportions of milk MUFA in organic farms when compared with higher intensity farms, although this was not consistent when comparisons between organic and conventional systems (organic medium intensity: 6.95% of DMI grazing vs. conventional low-input; 8.43% of DMI grazing [5]) were performed at an intensity level similar to the systems of this work (organic: 24.9% of offered DM grazing vs. conventional: 8.31% of offered DM grazing). Croissant et al. [7] found higher milk MUFA concentrations in cows fed pasture compared to TMR feeding [7], and this potentially explains the higher MUFA concentrations in low-input and organic milk when compared with conventional or high-intensity systems in previous studies [4,5]. The RDA results suggested that the concentrations of MUFA are not correlated to grazing intake in the present study and the major drivers were dry straights (positive correlation) and grass and wholecrop silages (negative correlation), with the former being higher in conventional diets and the latter higher in organic diets, thus providing a potential explanation for the higher MUFA content in the conventional milk in the present study. Butler et al. [4] also suggested that the higher inclusion of concentrates may reduce biohydrogenation, resulting in lower SFA but higher MUFA in milk from higher-intensity production systems.

The main MUFA in milk is OA, contributing 71% to the total MUFA in the present study. The concentrations of milk OA were higher in conventional milk when compared with organic milk. Previous investigations have found higher OA in organic and low-input milk when compared to conventional milk [5]. However, Ellis et al. [3] reported that conventional milk had higher concentrations of OA when compared to organic milk. The RDA in the current study suggests a positive association with dry straights, maize silage, and oil and that these dietary components are stronger drivers than that of grazing, which may explain the higher OA contents in the conventional milk. Vaccenic acid is also an important component of overall MUFAs, contributing 5.5% towards total MUFA content in the current study. The current study found that organic milk had higher concentrations of VA than conventional milk, which is consistent with other studies [4,5], while the pasture-based low-input farms (feeding > 95% DMI as fresh grass) produced milk with higher VA concentrations when compared with both conventional and organic milk [4]. Fresh grass is rich in ALNA and LA [38] biohydrogenation, within which, in the rumen, produces VA [39], thus explaining the higher concentration of milk VA in the organic herds with a higher grazing contribution to their diet. The positive correlation between grazing and VA is also supported by the RDA results in the present study. Additionally, the current study found no difference in *trans* FA between the production systems, but it was higher in conventional milk when VA was excluded. This suggests that a large proportion of *trans* FA in the organic milk may have been derived from biohydrogenation of LA and ALNA present in pasture than from VA [39].

Previous investigations have shown higher overall milk PUFA concentrations in organic and low-input milk compared with conventional and high-intensity [3,4,5], in agreement with the current study. Production system × month interaction was identified, with organic milk containing significantly higher PUFA during the grazing season (April–August), supporting previous suggestions that milk PUFA increased with grazing [3]; and is further supported by results from RDA in the present study. The dietary supply of higher amounts of unsaturated C18 FA, present in fresh herbage, increases the amounts of long-chained unsaturated FA transferring into milk and reduces short and medium-chain SFA synthesis [40].

Along with previous investigations [3,4,5], the n-3 concentration (primarily ALNA) in the present study was higher, and n-6 was lower in the organic (compared with conventional) milk, resulting in a lower n-6:n-3 ratio in the organic milk. These findings have been associated with the higher pasture intake in organic herds and the inclusion of clover in organic pastures and silages [3,5,41]. Organic farms in the current study grazed herds on pastures with varying grass:clover ratios, whereas conventional herds grazed mainly perennial ryegrass, thus explaining, in conjunction with the higher pasture intake, the higher n-3 (primarily represented by ALNA) and lower n-6:n-3 concentrations in organic milk, in agreement with previous studies [3,5,42]. The RDA also identified associations of increased grazing with higher n-3 and lower n-6:n-3 in milk. However, the RDA showed a negative association between grazing and ALNA but positive associations with oil, minerals, moist byproducts, and maize silage, which is possible if n-3 rich oil supplements (e.g., linseed; [43]) were used, although this information was not available.

Smaller concentrations of omega 3 FA in milk, such as with EPA and DHA in milk, can be obtained directly from the diet or synthesized endogenously via rumen microbes [44,45], although the extent of transfer from dietary sources to milk is limited due to extensive rumen biohydrogenation [45]. The organic milk in the current study had higher concentrations of EPA and DHA than the conventional milk, which is in agreement with previous investigations [26], supporting the theory that increasing pasture intake results in higher EPA and DHA milk concentrations [44]. This is also supported by the RDA results, showing positive correlations between grazing and EPA and DHA concentrations.

### 4.4. Nutritional Implications for Organic Milk Consumers

The individual SFAs C12:0, C14:0, and C16:0 are considered detrimental to human health, increasing the concentration of serum low-density lipoprotein (LDL) cholesterol [46]. However, a recent meta-analysis [47] observed reduced serum total:high-density lipoprotein (HDL) cholesterol (associated with a protective effect) with intakes of C12:0 and C14:0, suggesting that these individual FAs may not be as damaging as previously understood. Furthermore, Givens [46] proposed that the modification of an FA profile should, in fact, focus on a reduction in C16:0, for which, in the current study, we detected no difference between the systems. The latest UK National Diet and Nutrition survey [48] reports dairy fat intakes of 13.4 g/day for children 1.5–3.0 years of age, 9.8 g/day for children 4–10 years of age, 8.3 g/day for adolescents 11–18 years of age, 8.8 g/day for adults 19–64 years of age, 9.8 g/day for adults 65–74 years of age, and 10.6 g/day for adults 75+ years of age. Based on recorded energy intakes [48] and maximum dietary recommended values (DRV) for SFA intakes (<10% total energy intake) [10], consuming organic milk instead of conventional milk would increase the contribution of SFA from dairy fats (relative to overall max DRV) from 55.5% to 56.7% in children, from 28.5% to 29.1% in adolescents, and from 32.9% to 33.6% in adults. Based on this, and coupled with the lack of difference in milk C16:0 proportions between the systems, and the fact that the difference in SFA content between organic and conventional milk is restricted only to four months across the year (September–December), there are no nutritional implications for the consumers of organic milk. Consuming organic milk instead of conventional milk would decrease the contribution of MUFA from dairy fats (relative to overall DRV) from 19.5% to 18.0% for children, from 9.8% to 9.2% for adolescents, and from 11.3% to 10.7% for adults, and these differences are too small to incur any effects on consumers’ nutrition and health.

Vaccenic acid has been associated with some direct health benefits and is also the precursor for RA in the human body, which is associated with desirable effects on cardiovascular health [49]. *Trans* FAs are associated with increases in LDL cholesterol [50], but the impact of *trans* FA from animal-derived foods, particularly VA on human health has been disputed [47,50]. Consuming organic milk instead of conventional milk would decrease the contribution of *trans* FA from dairy fats (relative to overall DRV) from 16.0% to 9.0% in children, from 8.2% to 4.6% in adolescents, and from 8.7% to 5.3% in adults. These differences are too small to be associated with any potential health implications to consumers, also considering that these values include VA, a *trans* FA, which other studies have associated with neutral or beneficial effects on human health [51]. Studies have suggested that the replacement of SFA with *cis* PUFA in milk may have beneficial impacts on vascular health [40], but *cis* PUFA were not different between the organic and conventional milk in the present study.

The predominant n-3 in milk is ALNA, which was higher in organic herds than conventional herds and contributed towards higher overall n-3 content. Since ALNA cannot be synthesized in the human body, it is an essential FA [45] and is associated with several health benefits and metabolic functions in humans [52]. Consuming organic milk instead of conventional milk would increase the contribution of ALNA from dairy fats (relative to overall DRV) from 18.5% to 28.2% in children, from 9.5% to 14.5% in adolescents, and from 11% to 16.8% in adults. While EPA and DHA are considered to play essential roles and functions within the human body [53], and their intake from the diet is necessary due to inefficient synthesis from ALNA in humans [54], their concentration levels in milk are low. Consuming organic milk instead of conventional milk would increase the contribution of EPA+DHA from dairy fats (relative to overall DRV) from 2.1% to 3.2% in children, from 1.5% to 2.3% in adolescents, and from 1.7% to 2.7% in adults. Therefore, consuming organic milk instead of conventional milk is unlikely to result in significant increases in total ALNA, EPA, and DHA intakes, which is also in line with previous studies [45].

## 5. Conclusions

The conventional herds had higher yields (milk, ECMY, fat, and protein) and concentrations of protein and casein when compared with organic. Additionally, they were more efficient at converting feed to milk, fat, and protein. However, the organic herds produced more milk, fat, and protein per kg of non-grazing and concentrate ingredients due to a higher reliance on grazing, particularly during the outdoor grazing period. Interestingly, although the organic herds did not use antibiotics, the cases of mastitis were lower than in the conventional herds, potentially as a result of hygiene, environment, practice, and the breeds used. The organic milk contained more total SFA, mainly during the indoor housed period, originating from higher nutritionally beneficial C4:0–C8:0, rather than the nutritionally undesirable C16:0. Although, C14:0 was also higher in the organic milk. The organic milk had higher nutritionally desirable n-3, *cis* n-3 PUFA, EPA, and DHA and a lower n-6:n-3 ratio, probably a consequence of the higher grazing intake and clover contribution in pastures and silages. However, the FA differences were relatively small when intakes of dairy fat are considered, and consuming organic over conventional milk is unlikely to impact consumers’ nutrition. Therefore, dairy systems focusing on reducing external inputs (concentrate feeds) can benefit from increased grazing, when available, and improve efficiency without negatively impacting the FA profile or cow health.

## Data Availability

Restrictions apply to the availability of the data used for this study and data sharing is not applicable.

## Supplementary Materials

The following supporting information can be downloaded at: https://www.mdpi.com/article/10.3390/foods12081589/s1. Table S1. Mean, minimum and maximum values for the breed and diet composition of herds (%) from 67 farms in Southern England. Table S2. Means ± SE and P-values for the breed and diet composition of herds (%) from 67 Farms with two differing production systems (Conventional and organic) in Southern England between January and December (2019). Table S3. Means ± SE and P-values for the Productivity, basic composition, efficiency, health parameters and indicators of herds from 67 farms with two differing production systems (Conventional and organic) in Southern England between January and December (2019). Table S4. Means ± SE and P-values for the effect of production system (conventional, 41; Organic, 26) on the fatty acid profile of milk collected from 67 Farms across southern England. Table S5. Means ± SE and P-values for the FA profile of milk from 67 herds with two differing production systems (Conventional and organic) in Southern England between January and December (2019). Table S6. Means ± SE and P-values for the FA profile of milk from 67 farms with two differing production systems (Conventional and organic) in Southern England between January and December (2019). Figure S1: Gas chromatogram illustrating the separation of fatty acid methyl esters in milk from the bulk tanks of 67 farms across Southern England over 12 months. Figure S2: Individual saturated fatty acids not displayed in main text, where a significant (*p* < 0.05) interaction between production system and month was observed. Figure S3: Individual polyunsaturated fatty acids not displayed in main text, where a significant (*p* < 0.05) interaction between production system and month was observed. Figure S4: FA groups not displayed in main text, where a significant (*p* < 0.05) interaction between production system and month was observed.

## References

[1] Association S. Soil Association Standards Farming and Growing. https://www.soilassociation.org/media/15931/farming-and-growing-standards.pdf.

[2] Association S. Organic Market Report. https://www.soilassociation.org/certification/organic-market-report/.

[3] Ellis K.A., Innocent G., Grove-White D., Cripps P., McLean W.G., Howard C.V., Mihm M. (2006). Comparing the fatty acid composition of organic and conventional milk. J. Dairy Sci..

[4] Butler G., Nielsen J.H., Slots T., Seal C., Eyre M.D., Sanderson R., Leifert C. (2008). Fatty acid and fat-soluble antioxidant concentrations in milk from high- and low-input conventional and organic systems: Seasonal variation. J. Sci. Food Agric..

[5] Stergiadis S., Leifert C., Seal C.J., Eyre M.D., Larsen M.K., Slots T., Nielsen J.H., Butler G. (2015). A 2-year study on milk quality from three pasture-based dairy systems of contrasting production intensities in Wales. J. Agric. Sci..

[6] Rodríguez-Bermúdez R., Fouz R., Miranda M., Orjales I., Minervino A.H.H., López-Alonso M. (2019). Organic or conventional dairy farming in northern Spain: Impacts on cow reproductive performance. Reprod. Domest. Anim..

[7] Croissant A.E., Washburn S.P., Dean L.L., Drake M.A. (2007). Chemical Properties and Consumer Perception of Fluid Milk from Conventional and Pasture-Based Production Systems. J. Dairy Sci..

[8] Davis H., Stergiadis S., Chatzidimitriou E., Sanderson R., Leifert C., Butler G. (2020). Meeting Breeding Potential in Organic and Low-Input Dairy Farming. Front. Vet. Sci..

[9] Stergiadis S., Leifert C., Seal C.J., Eyre M.D., Nielsen J.H., Larsen M.K., Slots T., Steinshamn H., Butler G. (2012). Effect of Feeding Intensity and Milking System on Nutritionally Relevant Milk Components in Dairy Farming Systems in the North East of England. J. Agric. Food Chem..

[10] SACN Saturated Fats and Health. https://assets.publishing.service.gov.uk/government/uploads/system/uploads/attachment_data/file/814995/SACN_report_on_saturated_fat_and_health.pdf.

[11] Gaudaré U., Pellerin S., Benoit M., Durand G., Dumont B., Barbieri P., Nesme T. (2021). Comparing productivity and feed-use efficiency between organic and conventional livestock animals. Environ. Res. Lett..

[12] Brito A.F., Silva L.H.P. (2020). Symposium review: Comparisons of feed and milk nitrogen efficiency and carbon emissions in organic versus conventional dairy production systems. J. Dairy Sci..

[13] AHDB Is All-Year-Round Calving Really the Best Option. https://ahdb.org.uk/news/is-all-year-round-calving-really-the-best-option.

[14] Qin N., Faludi G., Beauclercq S., Pitt J., Desnica N., Pétursdóttir Á., Newton E.E., Angelidis A., Givens I., Juniper D. (2021). Macromineral and trace element concentrations and their seasonal variation in milk from organic and conventional dairy herds. Food Chem..

[15] Chilliard Y., Martin C., Rouel J., Doreau M. (2009). Milk fatty acids in dairy cows fed whole crude linseed, extruded linseed, or linseed oil, and their relationship with methane output1. J. Dairy Sci..

[16] Ulberth F., Gabernig R.G., Schrammel F. (1999). Flame-ionization detector response to methyl, ethyl, propyl, and butyl esters of fatty acids. J. Amer. Oil Chem. Soc..

[17] Mierlita D. (2018). Effects of diets containing hemp seeds or hemp cake on fatty acid composition and oxidative stability of sheep milk. S. Afr. J. Anim..

[18] Stergiadis S., Berlitz C.B., Hunt B., Garg S., Ian Givens D., Kliem K.E. (2019). An update to the fatty acid profiles of bovine retail milk in the United Kingdom: Implications for nutrition in different age and gender groups. Food Chem..

[19] VSN International (2020). Genstat for Windows.

[20] (2012). Canoco5.

[21] Stergiadis S., Bieber A., Chatzidimitriou E., Franceschin E., Isensee A., Rempelos L., Baranski M., Maurer V., Cozzi G., Bapst B. (2018). Impact of US Brown Swiss genetics on milk quality from low-input herds in Switzerland: Interactions with season. Food Chem..

[22] Średnicka-Tober D., Barański M., Seal C., Sanderson R., Benbrook C., Steinshamn H., Gromadzka-Ostrowska J., Rembiałkowska E., Skwarło-Sońta K., Eyre M. (2016). Composition differences between organic and conventional meat: A systematic literature review and meta-analysis. Br. J. Nutr..

[23] Kay J.K., Mackle T.R., Auldist M.J., Thomson N.A., Bauman D.E. (2004). Endogenous Synthesis of *cis-9*, *trans*-11 Conjugated Linoleic Acid in Dairy Cows Fed Fresh Pasture. J. Dairy Sci..

[24] Adler S.A., Jensen S.K., Govasmark E., Steinshamn H. (2013). Effect of short-term versus long-term grassland management and seasonal variation in organic and conventional dairy farming on the composition of bulk tank milk. J. Dairy Sci..

[25] White S.L., Bertrand J.A., Wade M.R., Washburn S.P., Green J.T., Jenkins T.C. (2001). Comparison of Fatty Acid Content of Milk from Jersey and Holstein Cows Consuming Pasture or a Total Mixed Ration. J. Dairy Sci..

[26] Schwendel B.H., Wester T.J., Morel P.C.H., Tavendale M.H., Deadman C., Shadbolt N.M., Otter D.E. (2015). Invited review: Organic and conventionally produced milk—An evaluation of factors influencing milk composition. J. Dairy Sci..

[27] Lorenz H., Reinsch T., Hess S., Taube F. (2019). Is low-input dairy farming more climate friendly? A meta-analysis of the carbon footprints of different production systems. J. Clean. Prod..

[28] Ellis K.A., Innocent G.T., Mihm M., Cripps P., McLean W.G., Howard C.V., Grove-White D. (2007). Dairy cow cleanliness and milk quality on organic and conventional farms in the UK. J. Dairy Res..

[29] Ward W.R., Hughes J.W., Faull W.B., Cripps P.J., Sutherland J.P., Sutherst J.E. (2002). Observational study of temperature, moisture, pH and bacteria in straw bedding, and faecal consistency, cleanliness and mastitis in cows in four dairy herds. Vet. Rec..

[30] Prendiville R., Pierce K.M., Buckley F. (2010). A comparison between Holstein-Friesian and Jersey dairy cows and their F1 cross with regard to milk yield, somatic cell score, mastitis, and milking characteristics under grazing conditions. J. Dairy Sci..

[31] Friggens N.C., Ridder C., Løvendahl P. (2007). On the Use of Milk Composition Measures to Predict the Energy Balance of Dairy Cows. J. Dairy Sci..

[32] Cabezas-Garcia E.H., Gordon A.W., Mulligan F.J., Ferris C.P. (2021). Revisiting the Relationships between Fat-to-Protein Ratio in Milk and Energy Balance in Dairy Cows of Different Parities, and at Different Stages of Lactation. Animals.

[33] Poljak F., Mijić P., Lončarić Z., Steiner Z., Gantner V. (2021). The analysis of variability of indicators associated with prevalence of subclinical ketosis/acidosis in dairy cattle. Agric. Conspec. Sci..

[34] Zhang R., Liu J., Jiang L., Mao S. (2020). Effect of high-concentrate diets on microbial composition, function, and the VFAs formation process in the rumen of dairy cows. Anim. Feed Sci. Technol..

[35] Schroeder G.F., Delahoy J.E., Vidaurreta I., Bargo F., Gagliostro G.A., Muller L.D. (2003). Milk Fatty Acid Composition of Cows Fed a Total Mixed Ration or Pasture Plus Concentrates Replacing Corn with Fat. J. Dairy Sci..

[36] Ormston S., Davis H., Butler G., Chatzidimitriou E., Gordon A.W., Theodoridou K., Huws S., Yan T., Leifert C., Stergiadis S. (2022). Performance and milk quality parameters of Jersey crossbreds in low-input dairy systems. Sci. Rep..

[37] Stergiadis S., Bieber A., Franceschin E., Isensee A., Eyre M.D., Maurer V., Chatzidimitriou E., Cozzi G., Bapst B., Stewart G. (2015). Impact of US Brown Swiss genetics on milk quality from low-input herds in Switzerland: Interactions with grazing intake and pasture type. Food Chem..

[38] Nguyen Q.V., Malau-Aduli B.S., Cavalieri J., Malau-Aduli A.E.O., Nichols P.D. (2019). Enhancing omega-3 long-chain polyunsaturated fatty acid content of dairy-derived foods for human consumption. Nutrients.

[39] Chilliard Y., Ferlay A., Mansbridge R.M., Doreau M. (2000). Ruminant milk fat plasticity: Nutritional control of saturated, polyunsaturated, trans and conjugated fatty acids. Ann. Zootech..

[40] Givens D.I. (2012). Milk in the diet: Good or bad for vascular disease?. Proc. Nutr. Soc..

[41] Nantapo C.T.W., Muchenje V., Hugo A. (2014). Atherogenicity index and health-related fatty acids in different stages of lactation from Friesian, Jersey and Friesian×Jersey cross cow milk under a pasture-based dairy system. Food Chem..

[42] Dewhurst R.J., Evans R.T., Scollan N.D., Moorby J.M., Merry R.J., Wilkins R.J. (2003). Comparison of Grass and Legume Silages for Milk Production. 2. In Vivo and In Sacco Evaluations of Rumen Function. J. Dairy Sci..

[43] Stergiadis S., Leifert C., Seal C.J., Eyre M.D., Steinshamn H., Butler G. (2014). Improving the fatty acid profile of winter milk from housed cows with contrasting feeding regimes by oilseed supplementation. Food Chem..

[44] Huang G., Zhang Y., Xu Q., Zheng N., Zhao S., Liu K., Qu X., Yu J., Wang J. (2020). DHA content in milk and biohydrogenation pathway in rumen: A review. PeerJ.

[45] Givens I.D., Gibbs R.A. (2008). Current intakes of EPA and DHA in European populations and the potential of animal-derived foods to increase them: Symposium on ‘How can the n-3 content of the diet be improved?. Proc. Nutr. Soc..

[46] Givens D.I. (2010). Milk and meat in our diet: Good or bad for health?. Animal.

[47] Mensink R.P., Zock P.L., Kester A.D., Katan M.B. (2003). Effects of dietary fatty acids and carbohydrates on the ratio of serum total to HDL cholesterol and on serum lipids and apolipoproteins: A meta-analysis of 60 controlled trials. Am. J. Clin. Nutr..

[48] England P.H. https://assets.publishing.service.gov.uk/government/uploads/system/uploads/attachment_data/file/943114/NDNS_UK_Y9-11_report.pdf.

[49] Jahreis G., Dawczynski C., Givens D.I. (2020). Chapter 4—Trans and conjugated fatty acids in dairy products: Cause for concern?. Milk and Dairy Foods.

[50] Haug A., Høstmark A.T., Harstad O.M. (2007). Bovine milk in human nutrition—A review. Lipids Health Dis..

[51] Verneque B.J.F., Machado A.M., de Abreu Silva L., Lopes A.C.S., Duarte C.K. (2022). Ruminant and industrial trans-fatty acids consumption and cardiometabolic risk markers: A systematic review. Crit. Rev. Food Sci. Nutr..

[52] Givens D.I. (2015). Manipulation of lipids in animal-derived foods: Can it contribute to public health nutrition?. Eur. J. Lipid Sci. Technol..

[53] Lavie Carl J., Milani Richard V., Mehra Mandeep R., Ventura Hector O. (2009). Omega-3 Polyunsaturated Fatty Acids and Cardiovascular Diseases. J. Am. Coll. Cardiol..

[54] Burdge G.C., Jones A.E., Wootton S.A. (2002). Eicosapentaenoic and docosapentaenoic acids are the principal products of alpha-linolenic acid metabolism in young men. Br. J. Nutr..

