# Supplementary material for: Implications of Organic Dairy Management on Herd Performance and Milk Fatty Acid Profiles and Interactions with Season
Source: Foods. 2023 Apr 8;12(8):1589. doi: 10.3390/foods12081589 (PMC10138061; doi:10.3390/foods12081589)
Supplement: Supplementary file 1 [file foods-12-01589-s001.zip › foods-2301152-supplementary.pdf]

Supplementary Data

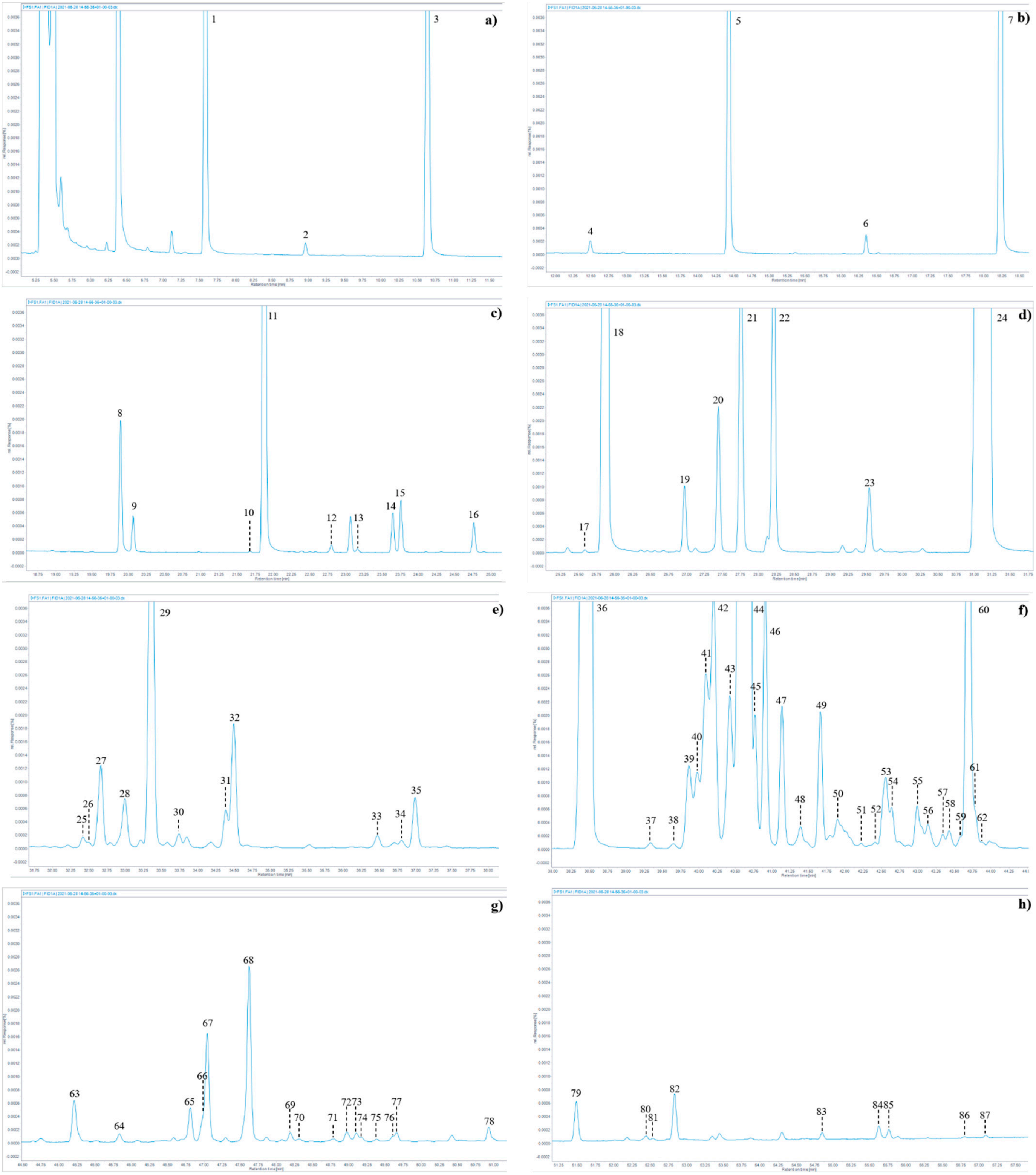

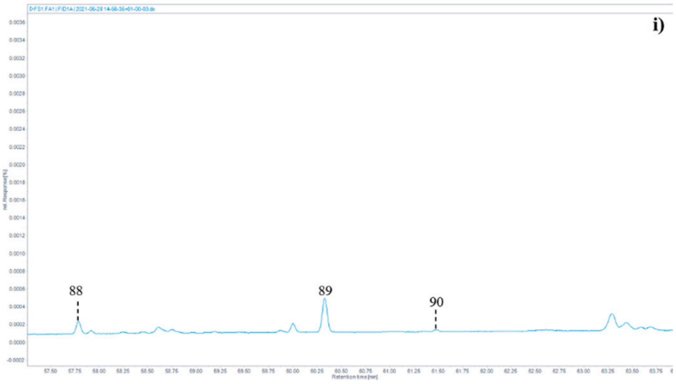

Figure S1: Gas chromatogram illustrating the separation of fatty acid methyl esters in milk from the bulk tanks of 67 farms across Southern England over 12 months. The left y-axis represents arbitrary response and the x-axis represents the retention time from 5 to 70 min. Chromatographic conditions are presented in the Materials and Methods. The following peaks were identified: 1=C4:0, 2=C5:0, 3=C6:0, 4=C7:0, 5=C8:0, 6=C9:0, 7=C10:0, 8=C10:1 *c*9, 9=C11:0, 10=C11:1 *c*10 11=C12:0, 12= C13:0 iso, 13=C13:0 anteiso, 14=C12:1 *c*9, 15=C13:0, 16=C14:0 iso, 17=C13:1, 18=C14:0, 19=C14:1 *t*9, 20=C15:0, 21=C15:1 *c*9, 22=C15:0 anteiso, 23=C16:0 iso, 24=C16:0, 25=C16:1 *t*6-8, 26=C16:1 *t*9, 27=C17:0 iso, 28=C16:1 *t*11/13, 29=C16:1 + C17:0 anteiso, 30=C16:1 *c*11, 31=C16:1 *c*13, 32=C17:0, 33=C17:1 *t*10, 34=C18:0 iso, 35=C17: *c*9, 36=C18:0, 37=C18:1 *t*4, 38=C18:1 *t*5, 39=C18:1 *t*6-8, 40=C18:1 *t*9, 41=C18:1 *t*10, 42=C18:1 *t*11, 43=C18:1 *c*6/*t*12, 44=C18:1 *c*9, 45=C18:1 *t*15, 46=C18:1 *c*11, 47=C18:1 *c*12, 48=C18:1 *c*13, 49=C18:1 *t*6 + C18:1 *c*14, 50=C19:0 + C18:1 *c*15, 51=C18:2 *t*11*t*15, 52=C18:2 *t*9*t*12, 53=C18:2 *c*8*c*13, 54=C18:2 *c*10*t*14, 55=C18:2 *c*9*c*14, 56=C18:2 *c*9*t*12, 57=C18:1 *c*16, 58=C18:1 *t*11*c*15, 59=C18:2 *t*9*c*12, 60=C18:2 *c*9*c*12, 61=C18:2 *t*12 *c*15, 62=C19:1, 63=C20:0, 64=C18:3 *c*6 *c*9 *c*12, 65=C20:1 *c*8, 66=C20:1 *c*11, 67=C18:3 *c*9 *c*12 *c*15, 68=CLA *c*9 *t*11+*t*7*c*9 +*t*8*c*10+*t*6*c*8, 69-76=other CLA of unknown isomerism, 77=C20:2 *c*11*c*14, 78=C22:0, 79=C20:3 *c*8*c*11*c*14, 80=C22:1 *c*13, 81=C20:3 *c*11*c*14*c*17, 82=C20:4 *c*5*c*8*c*11*c*14, 83=C22:2 *c*13*c*16, 84=C20:5 *c*5*c*8*c*11*c*14*c*17 (EPA), 85=C24:0, 86=C24:1 *c*15, 87=C22:3 *c*13*c*16*c*19, 88=C22:4 *c*7*c*10*c*13*c*16, 89=C22:5 *c*7*c*10*c*13*c*16*c*19 (DPA), 90=C22:6 *c*4*c*7*c*10*c*13*c*16*c*19 (DHA).

Table S1. Mean, minimum and maximum values for the breed and diet composition of herds (%) from 67 farms in Southern England.

	Mean n=67	Min n=67	Max n=67	SE
Milking herd size (number of cows)	247	75	670	4.0
Milking cows (% herd)	86	30	100	0.4
Estimated Liveweight (kg)[1]	649	468	680	1.7
Holstein	74.8	0	100	1.36
British Friesian	3.28	0	100	0.493
NZ Friesian	0.56	0	100	0.257
Jersey	0.53	0	16.3	0.079
Scandinavian Red	0.42	0	41.0	0.070
Shorthorn	0.30	0	6.67	0.036
Ayrshire	4.76	0	100	0.723
Montbeliarde	0.06	0	6.45	0.017
Brown Swiss	0.95	0	51.4	0.192
Guernsey	0.03	0	1.57	0.007
Other breed or crossbreed	14.3	0	100	1.19
Offered feed (kg DM / day)[2]	20.2	6.80	31.7	0.09
Total Forage	66.2	39.4	95.6	0.40
Total concentrate	33.9	4.37	60.6	0.40
Predicted grazing intake	14.8	0	91.4	0.84
Total Silage intake	49.3	0	114	0.69
Grass silage	26.9	0	81.9	0.62
Grass:clover silage[4]	1.77	0	67.3	0.246
Maize silage	15.6	0	54.2	0.54
Cereal silage	0.15	0	35.9	0.066
Lucerne silage	12.5	0.25	0	0.05
Other mixed silage	53.7	1.12	0	0.17
Wholecrop	49.8	3.49	0	0.28
Hay and Straw	30.4	2.26	0	0.12
Moist by-products	25.6	3.03	0	0.19
Dry straights[3]	44.4	6.77	0	0.40
Cereals	21.0	3.06	0	0.18
Blends	59.1	20.5	0	0.45
Oil	4.03	0.51	0	0.029
Minerals (g/cow per day)	750	110	0	3.9
Vitamins (g/cow per day)	320	6.59	0	1.4

[1]Average herd live weights were estimated based on average breed live weights and the proportionate number of cows from each breed or crossbreed in the total herd, as described by Stergiadis, et al. [1].

[2]When cows had no access to pasture, this value reflects the summary of individual feeds DM as recorded in farmers' questionnaires. During the grazing season, this is predicted using equations published by [2].

[3]Dry straights: single feeding stuffs which from which compound feeds and protein concentrates are prepared (wheat, barley, flaked maize, field beans, groundnut cake and meal, soya bean meal).

Table S2. Means ± SE and P-values for the breed and diet composition of herds (%) from 67 Farms with two differing production systems (Conventional and organic) in Southern England between January and December (2019)

	Month													
	January n=65[1]	February n=65[1]	March n=66[1]	April n=67[1]	May n=67[1]	June n=67[1]	July n=67[1]	August n=67[1]	September n=67[1]	October n=66[1]	November n=65[1]	December n=65[1]	SE	P-Value[2]
Milking herd size (no.cows)	244^{bcd}	240^{bcd}	251^{a}	254^{a}	253^{a}	250^{ab}	240^{cd}	236^{d}	245^{abc}	249^{ab}	249^{ab}	248^{abc}	14.0	<0.001
Milking cows (% herd)	85.5^{defg}	84.7^{efgh}	86.8^{abcdf}	87.6^{abc}	87.9^{ac}	86.3^{bcdef}	83.4^{gh}	82.6^{h}	85^{fg}	85.9^{cdef}	86.7^{abcde}	87.0^{abcde}	1.21	0.002
Estimated Liveweight (kg)[3]	653	650	650	650	650	650	650	650	647	645	646	647	6.0	0.567
Breed composition (% of herd)														
Holstein	78.2	75.7	75.1	75.1	75.3	75.3	75.4	75.2	73.9	72.5	72.6	73.6	4.75	0.523
British Friesian	2.53	2.51	3.11	3.11	2.93	3.08	3.05	3.05	2.91	4.28	4.30	4.49	1.707	0.333
NZ Friesian	0.75	0.00	0.00	0.00	0.00	0.00	0.00	0.00	1.49	1.49	1.49	1.54	0.564	0.462
Jersey	0.51	0.49	0.44	0.48	0.61	0.50	0.46	0.58	0.59	0.56	0.56	0.59	0.272	0.478
Scandinavian Red	0.89	0.20	0.44	0.41	0.40	0.38	0.41	0.45	0.38	0.38	0.37	0.40	0.211	0.353
Shorthorn	0.26	0.24	0.38	0.42	0.36	0.29	0.29	0.27	0.25	0.24	0.26	0.28	0.125	0.213
Ayrshire	4.91	4.75	4.66	4.67	4.76	4.76	4.76	4.76	4.76	4.76	4.74	4.89	2.523	0.997
Montbeliarde	0.13	0.10	0.13	0.13	0.03	0.03	0.01	0.01	0.02	0.02	0.02	0.05	0.045	0.405
Brown Swiss	0.39	0.38	1.01	1.01	1.01	1.04	1.07	1.13	1.08	1.12	1.11	1.06	0.639	0.505
Guernsey	0.03	0.03	0.03	0.03	0.03	0.03	0.03	0.03	0.03	0.03	0.03	0.04	0.025	0.924
Other breed or crossbreed	11.4	15.6	14.7	14.7	14.6	14.6	14.5	14.6	14.6	14.6	14.5	13.0	4.14	0.178
Diet Composition (% DM offered unless otherwise stated)														
Offered feed (kg DM / day)[4]	20.7^{abc}	20.7^{ab}	20.9^{a}	20.4^{bcd}	20.2^{bcd}	19.9^{cde}	19.8^{de}	19.8^{cde}	19.8^{de}	20.1^{bcd}	19.4^{e}	20.5^{bcd}	0.288	<0.001
Total Forage	64.7	64.5	64.6	66.4	66.7	66.9	67.9	68.2	66.1	66.1	65.8	65.8	1.39	0.108
Total concentrate	35.4	35.6	35.4	33.6	33.3	33.1	32.1	31.8	33.9	33.9	34.2	34.2	1.39	0.108
Predicted grazing intake	0.00^{f}	0.00^{f}	6.40^{e}	25.2^{cb}	30.9^{a}	31.3^{a}	29.6^{ba}	24.8^{c}	19.3^{d}	9.09^{e}	0.00^{f}	0.00^{f}	1.928	<0.001
Total Silage intake	61.3^{a}	61.1^{a}	56.0^{b}	39.2^{ed}	33.8^{g}	33.7^{g}	35.7^{eg}	38.9^{d}	41.5^{c}	55.5^{b}	64.7^{a}	64.1^{a}	1.94	<0.001
Grass silage	33.5^{ab}	33.^{2ab}	29.6^{cd}	20.0^{e}	16.0^{f}	16.6^{f}	20.2^{e}	25.0^{d}	25.2^{d}	30.9^{bc}	36.9^{a}	36.6^{a}	1.94	<0.001
Grass:clover silage[5]	3.63^{ab}	3.88^{a}	4.04^{a}	0.55^{c}	0.68^{c}	0.76^{c}	0.82^{c}	0.76^{c}	1.04^{c}	1.20^{c}	2.10^{bc}	1.80^{bc}	0.741	<0.001
Maize silage	17.6^{ab}	17.9^{ab}	16.6^{ba}	15.2^{fdba}	14.9^{gfed}	13.9^{efgh}	13.8^{gh}	12.5^{h}	14.0^{fgh}	15.5^{gedcb}	17.^{5acd}	17.4^{acd}	1.87	<0.001

Cereal silage	0.00	0.00	0.00	0.00	0.00	0.00	0.00	0.00	0.00	2.00	1.76	0.56	0.271	0.086
Lucerne silage	0.33^{abcd}	0.27^{abcd}	0.24^{cd}	0.25^{abcd}	0.20^{a}	0.17^{d}	0.17^{d}	0.17^{d}	0.17^{d}	0.19^{d}	0.26^{bcd}	0.60^{ac}	0.161	0.027
Other mixed silage	0.89	1.26	1.14	1.12	0.89	1.14	0.77	0.47	1.10	0.59	1.48	2.66	0.542	0.200
Wholecrop	5.28^{dcba}	4.53^{cd}	4.39^{cd}	2.10^{fg}	1.12^{gh}	1.14^{gh}	0.43^{h}	2.56^{fg}	3.40^{df}	6.28^{a}	5.85^{ac}	4.84^{bcd}	0.895	<0.001
Hay and Straw	3.37^{a}	3.39^{a}	2.21^{b}	2.01^{b}	2.04^{b}	1.99^{b}	2.20^{b}	1.94^{b}	1.93^{b}	2.38^{b}	1.75^{b}	1.95^{b}	0.387	0.035
Moist by-products	3.02	3.13	3.32	2.91	2.92	3.16	3.25	2.98	3.18	2.8	3.09	2.62	0.654	0.359
Dry straights[6]	8.31	8.03	7.37	6.77	6.31	6.60	6.04	5.81	6.52	6.92	6.50	6.13	1.384	0.608
Cereals	3.67^{abcd}	3.36^{abcd}	3.56^{ac}	2.68^{bcd}	2.83^{abcd}	2.30^{d}	2.80^{abcd}	3.17^{abcd}	3.06^{abcd}	3.11^{abcd}	2.33^{cd}	3.90^{ab}	0.609	0.029
Blends	19.9	20.5	20.7	20.8	20.7	20.5	19.5	19.3	20.6	20.6	21.8	21.0	1.55	0.456
Oil	0.49	0.50	0.48	0.53	0.48	0.50	0.53	0.53	0.54	0.50	0.50	0.56	0.102	0.830
Minerals (g/cow per day)	112	116	111	113	100	95.4	98.6	101	106	120	118	123	13.3	0.236
Vitamins (g/cow per day)	8.80^{abc}	8.60^{abc}	8.60^{abc}	7.56^{abc}	7.39^{abc}	7.43^{abc}	6.57^{abc}	6.57^{abc}	6.57^{ba}	0.00^{c}	0.00^{cb}	11.2^{a}	4.40	0.001

[1]n is the number of records used to calculate means ± SE and P-values. Records with missing values were not included in the analysis.

[2]Significances were declared at P-value <0.05.

[3]Average herd live weights were estimated based on average breed live weights and the proportionate number of cows from each breed or crossbreed in the total herd, as described by Stergiadis, et al. [1].

[4]When cows had no access to pasture, this value reflects the summary of individual feeds DM as recorded in farmers' questionnaires. During the grazing season, this is predicted using equations published by Butler et al. (2008)

[5]In the conventional production system, this was predominantly perennial ryegrass silage, while in organic systems silage typically has variant grass:clover ratios.

[6]Dry straights: single feeding stuffs which from which compound feeds and protein concentrates are prepared (wheat, barley, flaked maize, field beans, groundnut cake and meal, soya bean meal).

Table S3. Means ± SE and P-values for the Productivity, basic composition, efficiency, health parameters and indicators of herds from 67 farms with two differing production systems (Conventional and organic) in Southern England between January and December (2019)

	Month													
	January n=65[1]	February n=67[1]	March n=67[1]	April n=67[1]	May n=67[1]	June n=67[1]	July n=67[1]	August n=67[1]	September n=67[1]	October n=67[1]	November n=67[1]	December n=65[1]	SE	P-Value[2]
Productivity (kg/cow per day)														
Milk Yield	27.0^{bcd}	26.6^{d}	26.9^{d}	27.7^{ac}	27.8^{ac}	26.9^{bcd}	26.7^{d}	26.2^{d}	26.0^{d}	25.9^{d}	26.1^{d}	26.9^{cd}	0.71	0.007
ECMY	28.0^{abcdef}	27.4^{abcdef}	27.5^{bcdef}	28.0^{ac}	27.9^{ecba}	27.0^{cef}	26.1^{g}	26.7^{f}	26.8^{efg}	27.2^{cdef}	27.6^{abcde}	28.1^{abcd}	0.69	0.003
Milk fat yield	1.08^{ac}	1.05^{bcd}	1.06^{abcd}	1.06^{abc}	1.05^{cba}	1.02^{d}	1.00^{e}	1.02^{cd}	1.03^{cd}	1.05^{abcd}	1.07^{ab}	1.09^{ab}	0.026	<0.001
Milk protein yield	1.00^{abcde}	0.89^{bcde}	0.88^{de}	0.91^{ac}	0.91^{ac}	0.87^{de}	0.86^{e}	0.86^{e}	0.87^{e}	0.88^{cde}	0.90^{abd}	0.91^{abcd}	0.023	<0.001
Basic Composition (g/kg milk)														
Milk fat	40.3^{bcde}	39.2^{f}	39.5^{def}	38.5^{g}	37.7^{h}	38.2^{gh}	36.7^{i}	39.3^{ef}	39.9^{cdf}	40.9^{ab}	41.5^{a}	40.6^{bc}	0.32	<0.001
Milk protein	33.3^{cd}	33.2^{cd}	32.8^{efg}	33.0^{cd}	32.8^{def}	32.6^{f}	32.4^{g}	33.0^{cde}	33.4^{c}	34.1^{a}	34.7^{a}	34.0^{b}	0.19	<0.001
Milk casein	26.1^{ef}	26.0^{fgh}	25.7^{ghi}	25.8^{fgh}	25.8^{fg}	25.5^{hi}	25.4^{i}	26.1^{ef}	26.5^{ec}	27.1^{b}	27.7^{a}	26.8^{bc}	0.17	<0.001
Milk whey protein	7.18^{b}	7.28^{a}	7.08^{cd}	7.22^{ab}	7.06^{de}	7.10^{c}	6.99^{ef}	7.00^{de}	6.94^{f}	7.02^{de}	7.04^{cde}	7.24^{ab}	0.032	<0.001
Milk lactose	45.3^{bc}	45.2^{bcd}	45.5^{a}	45.3^{b}	45.2^{b}	45.1^{cd}	44.9^{f}	44.6^{g}	44.6^{g}	44.9^{ef}	45.0^{df}	45.0^{def}	0.09	<0.001
Urea	0.16^{d}	0.13^{fg}	0.14^{ef}	0.12^{h}	0.12^{gh}	0.11^{h}	0.15^{de}	0.14^{def}	0.23^{a}	0.20^{bc}	0.20^{b}	0.19^{c}	0.007	<0.001
Efficiency Parameters														
Feed efficiency (kg milk/kg DM offered)	1.31^{bcde}	1.30^{cde}	1.28^{e}	1.35^{abd}	1.38^{a}	1.34^{abcd}	1.34^{bcde}	1.31^{bcde}	1.31^{bcde}	1.28^{de}	1.35^{abc}	1.32^{abcde}	0.029	<0.001
Feed non-grazing efficiency (kg milk/kg non-grazing DM offered)	1.31^{e}	1.30^{e}	1.39^{e}	2.39^{b}	2.71^{a}	2.72^{a}	2.43^{b}	2.01^{c}	1.75^{d}	1.48d^{e}	1.35^{e}	1.32^{e}	0.117	<0.001
Feed concentrate efficiency (kg milk/kg concentrate DM offered)	4.21^{ef}	4.24^{fedc}	4.03^{f}	4.85^{abd}	4.91^{ab}	4.80^{efcba}	4.81^{bcde}	5.31^{a}	4.35^{bcdef}	4.31^{ef}	4.20^{ef}	4.28^{edf}	0.249	0.007
Fat efficiency (g fat/kg DM offered)	52.7^{bc}	51.2^{bcde}	50.4^{de}	52.1^{bc}	51.8^{bc}	51.1^{cd}	48.6^{e}	51.3^{bcd}	51.8^{bcd}	52.2^{bc}	55.8^{a}	53.2^{b}	1.01	<0.001
Fat non-grazing efficiency (g fat/kg non-grazing DM offered)	52.7^{e}	51.2^{e}	55.1^{e}	91.8^{b}	102^{a}	105^{a}	91.3^{b}	79.9^{c}	69.7^{d}	60.5^{e}	55.8^{e}	53.2^{e}	4.79	<0.001

Fat concentrate efficiency (g fat/kg concentrate DM offered)	170^{bc}	168^{bc}	160^{c}	187^{ab}	186^{b}	185^{b}	179^{bc}	212^{a}	174.1^{bc}	176^{bc}	175^{bc}	174^{bc}	10.4	0.027
Protein efficiency (g protein/kg DM offered)	43.6^{fgh}	43.4^{h}	42.0^{i}	44.7^{cdefg}	45.1^{dfcba}	43.7^{efgh}	42.9^{hi}	43.2^{ghi}	43.5^{fgh}	43.8^{defgh}	46.8^{ca}	44.6^{bcdefgh}	0.94	<0.001
Protein non-grazing efficiency (g protein/kg non-grazing DM offered)	43.6^{e}	43.4^{e}	45.8^{e}	79.4^{b}	89.6^{a}	90.5^{a}	81.1^{b}	67.4^{c}	58.3^{d}	50.6^{de}	46.8^{e}	44.6^{e}	4.16	<0.001
Protein concentrate efficiency (g protein/kg concentrate DM offered)	140^{bc}	143^{bc}	133^{c}	160^{ab}	162^{ab}	158^{b}	158^{b}	180^{a}	145^{bc}	147^{bc}	146^{bc}	145^{bc}	8.9	0.014
Health parameters and indicators														
Mastitis (% of herd)	2.99^{ab}	2.17^{cde}	2.53^{abc}	1.92^{de}	1.81^{e}	2.30^{cd}	2.23^{cde}	2.89^{ab}	2.55^{bcd}	2.69^{abc}	3.07^{a}	2.24^{cde}	0.216	<0.001
Lameness (% of herd)	2.58	2.23	2.02	2.52	2.19	2.11	2.57	2.36	2.38	2.46	2.05	1.96	0.296	0.803
Other disease (% of herd)	0.61^{bcd}	0.56^{cd}	0.57^{cd}	0.55^{cd}	0.63^{bcd}	0.63^{bcd}	0.83^{bcd}	1.16^{d}	0.80^{bcd}	0.94^{ab}	0.81^{abc}	0.38^{d}	0.132	0.032
Fat:protein	1.21^{a}	1.18^{bc}	1.21^{a}	1.17^{de}	1.15^{ef}	1.17^{dc}	1.14^{f}	1.19^{ba}	1.20^{ab}	1.20^{ab}	1.19^{ab}	1.19^{ab}	0.009	<0.001
Milk SCC (x1000/ml milk)	149^{cd}	129^{ef}	156^{abcd}	151^{cd}	141^{de}	165^{ac}	149^{bcd}	160^{abc}	114^{f}	143^{de}	142^{de}	138^{de}	6.7	<0.001

[1]n is the number of records used to calculate means ± SE and P-values. Records with missing values were not included in the analysis.

[2]Significances were declared at P-value <0.05.

Table S4. Means ± SE and P-values for the effect of production system (conventional, 41; Organic, 26) on the fatty acid profile of milk collected from 67 Farms across southern England

	Production System			
	Conventional	Organic		
	n[1]=485	n[1]=309	SE	P-value[2]
C4:0	26.2	26.8	0.08	<0.001
C5:0	0.26	0.24	0.003	<0.001
C6:0	18.5	19.3	0.06	<0.001
C7:0	0.25	0.23	0.004	<0.001
C8:0	11.6	12.0	0.05	0.029
C9:0	0.32	0.29	0.005	<0.001
C10:0	27.1	27.9	0.17	0.292
C10:1 *c*9	2.64	2.85	0.020	<0.001
C11:0	0.59	0.54	0.010	0.004
C11:1 *c*10	0.02	0.02	0.0004	0.056
C12:0	34.0	34.5	0.24	0.968
C13:0 iso	0.22	0.33	0.004	<0.001
C13:0 anteiso	0.09	0.11	0.001	<0.001
C12:1 *c*9	0.86	0.87	0.008	0.841
C13:0	0.95	0.97	0.011	0.661
c14:0 iso	0.75	1.08	0.009	<0.001
C13:1	0.05	0.05	0.001	0.942
C14:0	106	113	0.4	<0.001
C14:1 *t*9	1.97	2.65	0.020	<0.001
C15:0	4.15	4.87	0.0350	<0.001
C15:1 *c*9	9.45	9.57	0.063	0.434
C15:0 anteiso	10.2	11.9	0.08	<0.001
C16:0 iso	1.88	2.30	0.0170	<0.001
C16:0	314	309	1.5	0.905
C16:1 *t*6-8	0.31	0.27	0.002	<0.001
C16:1 *t*9	0.09	0.06	0.002	<0.001
C17:0 iso	3.47	4.05	0.036	<0.001
C16:1 *t*11/13	1.99	1.92	0.017	0.002
C16:1 + C17:0 anteiso	18.7	17.3	0.09	<0.001
C16:1 *c*11	0.93	1.17	0.018	<0.001
C16:1 *c*13	1.17	1.23	0.014	0.199
C17:0	4.90	5.96	0.028	<0.001
C17:1 *t*10	0.52	0.54	0.004	0.018
C18:0 iso	0.41	0.52	0.008	<0.001
C17:0 *c*9	1.97	2.28	0.014	<0.001
C18:0	99.2	102	0.59	0.080
C18:1 *t*4	0.20	0.13	0.003	<0.001
C18:1 *t*5	0.17	0.11	0.002	<0.001

C18:1 *t*6-8	3.37	2.37	0.033	<0.001
C18:1 *t*9	2.56	1.80	0.025	<0.001
C18:1 *t*10	5.79	3.93	0.105	<0.001
C18:1 *t*11	11.9	16.8	0.30	<0.001
C18:1 *c*6/*t*12	4.90	3.38	0.043	<0.001
C18:1 *c*9	198	185	0.9	<0.001
C18:1 *t*15	3.58	3.10	0.039	<0.001
C18:1 *c*11	6.87	4.39	0.057	<0.001
C18:1 *c*12	2.66	1.77	0.040	<0.001
C18:1 *c*13	0.73	0.57	0.009	<0.001
C18:1 *t*6 + C18:1 *c*14	3.60	3.23	0.026	<0.001
C19:0 + C18:1 *c*15	1.56	1.49	0.014	0.364
C18:2 *t*11*t*15	0.19	0.38	0.008	<0.001
C18:2 *t*9*t*12	0.05	0.03	0.001	<0.001
C18:2 *c*8*c*13	2.39	1.86	0.024	<0.001
C18:2 *c*10*t*14	1.11	1.12	0.014	0.727
C18:2 *c*9*c*14	1.40	1.07	0.010	<0.001
C18:2 *c*9*t*12	0.68	0.54	0.005	<0.001
C18:1 *c*16	0.31	0.30	0.006	0.408
C18:1 *t*11*c*15	1.32	2.16	0.042	<0.001
C18:2 *t*9*c*12	0.13	0.10	0.004	0.211
C18:2 *c*9*c*12	19.1	16.0	0.21	<0.001
C18:2 *t*12*c*15	0.88	0.97	0.014	<0.001
C19:1	0.17	0.22	0.003	<0.001
C20:0	1.49	1.87	0.012	<0.001
C18:3 *c*6*c*9*c*12	0.21	0.19	0.002	0.031
C20:1 *c*8	1.05	1.25	0.009	<0.001
C20:1 *c*11	0.54	0.56	0.013	0.076
C18:3 *c*9c12*c*15	4.53	6.94	0.063	<0.001
CLA c9 *t*11 + *t*7 *c*9 + *t*8 *c*10 + *t*6 *t*8	5.94	8.00	0.123	<0.001
CLA other a	0.22	0.30	0.002	<0.001
CLA other b	0.17	0.40	0.008	<0.001
CLA other c	0.14	0.17	0.002	<0.001
CLA other d	0.41	0.38	0.006	0.031
CLA other e	0.22	0.20	0.001	<0.001
CLA other f	0.05	0.04	0.001	<0.001
CLA other g	0.12	0.21	0.004	<0.001
CLA other h	0.11	0.16	0.002	<0.001
C20:2 *c*11*c*14	0.20	0.20	0.002	0.311
C22:0	0.51	0.85	0.007	<0.001
C20:3 *c*8*c*11*c*14	0.87	0.71	0.007	<0.001
C22:1 *c*13	0.16	0.55	0.023	<0.001
C20:3 *c*11*c*14*c*17	0.07	0.13	0.002	<0.001

C20:4 *c*5*c*8*c*11*c*14	1.11	0.96	0.009	<0.001
C22:2 *c*13*c*16	0.34	0.44	0.006	<0.001
C20:5 *c*5*c*8*c*11*c*14*c*17 (EPA)	0.44	0.66	0.006	<0.001
C24:0	0.35	0.52	0.004	<0.001
C24:1 *c*15	0.06	0.10	0.002	<0.001
C22:3 *c*13*c*16*c*19	0.08	0.12	0.003	<0.001
C22:4 *c*7*c*10*c*13*c*16	0.05	0.07	0.001	<0.001
C22:5 *c*7*c*10*c*13*c*16*c*19 (DPA)	0.74	1.05	0.007	<0.001
C22:6 *c*4 *c*7*c*10*c*13*c*16*c*19 (DHA)	0.05	0.07	0.001	<0.001

[1]n is the number of records used to calculate means. Records with missing data were not included in the means ± SE and P-values calculations.

[2]Significances were declared at P<0.05

Table S5. Means ± SE and P-values for the FA profile of milk from 67 herds with two differing production systems (Conventional and organic) in Southern England between January and December (2019)

	Month													
	January n=65[1]	February n=65[1]	March n=66[1]	April n=67[1]	May n=67[1]	June n=67[1]	July n=67[1]	August n=67[1]	September n=67[1]	October n=66[1]	November n=65[1]	December n=65[1]	SE	P-Value[2]
Individual FA (g/kg total FA)														
SFA														
C4:0	26.5[ba]	26.6[bc]	27.2[a]	26.6[b]	26.4[bc]	26.0[cd]	27.5[a]	27.1[a]	26.8[b]	25.4[e]	25.0[f]	25.7[de]	2.34	<0.001
C6:0	18.9[bc]	18.8[bc]	18.8[bc]	18.6[bc]	18.2[d]	17.9[e]	18.9[bc]	18.8[bc]	18.9[bc]	19.1b[ab]	19.0[b]	19.3[a]	1.65	<0.001
C8:0	11.8[bcdef]	11.7[bcdef]	11.6[cdef]	11.7[bd]	11.4[ef]	11.1[g]	11.5[f]	11.5[def]	11.7[bce]	12.3[a]	12.4[a]	12.4[a]	1.05	<0.001
C10:0	27.7[c]	27.4[cd]	26.9[de]	27.4[c]	27.0[cd]	25.7[e]	25.9[e]	26.0[e]	26.7[cd]	28.9[b]	29.6[a]	29.5[ab]	2.52	<0.001
C12:0	35.0[c]	34.5[c]	33.6[def]	34.0[c]	33.2[cde]	31.7[g]	32.2[fg]	32.6[efg]	33.6[cd]	36.3[b]	37.2[a]	36.8[ab]	3.20	<0.001
C14:0	112[cd]	110[d]	107[e]	105[g]	106[efg]	104[fg]	106[eg]	106[efg]	107[e]	113[bc]	115[a]	114[ab]	9.80	<0.001
C16:0	321[c]	315[d]	306[ef]	287[i]	295h[h]	300[g]	306[f]	313[de]	313[de]	330[ab]	334[a]	327[b]	28.2	<0.001
C18:0	98.5[e]	101[dc]	106[ba]	106[ab]	105[ab]	107[a]	103[cb]	99.9[ed]	99.2[ed]	92.8[gf]	91.4[g]	95.2[f]	10.16	<0.001
MUFA														
VA (C18:1 *t*11)	11.2[def]	11.4[def]	12.2[de]	19.3[a]	19.0[a]	16.1[b]	15.4[b]	13.6[c]	13.5[c]	11.8[d]	11.0[ef]	10.6[f]	1.90	<0.001
OA (C18:1 *c*9)	189[h]	191[hg]	197[fed]	204[ab]	202[bce]	206[a]	198[cdef]	198[ef]	196[gf]	181[i]	177[j]	179[ij]	19.2	<0.001
PUFA														
LA (18:2 *c*9*c*12)	18.4[cdef]	18.8[be]	19.2[a]	18.4[bce]	17.8[def]	17.8[def]	17.0[h]	16.0[gh]	17.5[fh]	17.5[fgh]	17.7[efh]	17.9[efgh]	2.00	<0.001
RA	5.59[de]	5.7[de]	5.80[de]	8.44[a]	8.80[a]	7.76[b]	7.56[bc]	7.27[c]	7.17[c]	5.95[d]	5.44[e]	5.24[e]	0.905	<0.001
ALNA (18:3 *c*9*c*12*c*15)	4.68[fe]	4.92[df]	5.11[d]	6.60[a]	6.08[b]	6.06[b]	6.01[b]	6.01[b]	5.64[c]	4.91[def]	4.86[efd]	4.66[f]	0.692	0.003
EPA (20:5 *c*5*c*8*c*11*c*14*c*17)	0.42[h]	0.47[g]	0.47[g]	0.55[cd]	0.57[b]	0.52[f]	0.60[a]	0.57[bc]	0.58[b]	0.54[d]	0.51[e]	0.49[fg]	0.061	<0.001
DPA (C22:5 *c*7*c*10*c*13*c*16*c*19)	0.79[d]	0.79[d]	0.76[e]	0.79[d]	0.90[b]	0.91[b]	0.96[a]	0.91[b]	0.92[b]	0.90[b]	0.87[c]	0.84[c]	0.100	<0.001
DHA(c22:6 *c*4*c*7*c*10*c*13*c*16*c*19)	0.05[f]	0.06[fe]	0.05[gh]	0.04[h]	0.05[g]	0.04[gh]	0.06[ce]	0.06[ef]	0.07[ab]	0.07[ab]	0.07[a]	0.07[ab]	0.007	0.001
FA groups (g/kg total FA)														
SFA[3]	694[c]	689[d]	679[e]	659[g]	665[f]	667[f]	676[e]	679[e]	680[ed]	701[b]	707[a]	704[ab]	58.9	0.001
MUFA[4]	264[e]	268[d]	277[c]	292[a]	287[b]	287[b]	279[c]	277[c]	276[cd]	257[f]	251[g]	255[gf]	26.6	<0.001
*cis*MUFA[6]	231[e]	234[de]	240[bc]	247[a]	244[b]	247[a]	240[cbd]	240[cbd]	239[cd]	224[f]	220[g]	222[fg]	22.8	<0.001

*trans*MUFA[7]	32.5^{fgh}	34.4^{f}	37.1^{d}	45.0^{a}	43.4^{b}	39.6^{c}	39.1^{c}	36.7^{d}	36.8^{d}	32.9^{gf}	31.3^{h}	32.3^{hg}	4.11	<0.001
PUFA[5]	42.2^{gh}	43.7^{eg}	44.8^{de}	52.9^{a}	52.1^{a}	48.8^{b}	47.5^{bc}	47.1^{cd}	45.81^{de}	41.7^{gh}	40.3^{h}	41.1^{h}	0.69	<0.001
*cis*PUFA[8]	27.3^{cde}	28.0^{bde}	28.4^{ab}	29.2^{a}	28.1^{b}	28.1^{bc}	27.7^{bcde}	27.3^{bcde}	27.5^{bcd}	26.8^{e}	27.0^{de}	27.0^{de}	0.54	<0.001
*trans*PUFA[9]	0.22^{fg}	0.24^{fg}	0.26^{ef}	0.50^{a}	0.47^{b}	0.37^{c}	0.34^{d}	0.33^{d}	0.30^{e}	0.24^{f}	0.21^{g}	0.21^{fg}	0.048	<0.001
cis,trans + *trans,cis*PUFA[10]	14.5^{def}	14.7^{de}	15.1^{d}	19.4^{a}	19.5^{a}	17.7^{b}	17.5^{b}	17.0^{bc}	16.4^{c}	14.7^{d}	14.0^{ef}	13.7^{f}	0.41	<0.001
n-3[11]	8.55^{gh}	8.66^{gh}	9.24^{f}	12.0^{a}	11.3^{bd}	10.9^{cd}	11.3^{bc}	10.9^{d}	10.2^{e}	9.00^{fg}	8.60^{h}	8.39^{h}	0.347	<0.001
n-6[12]	31.8	31.7	31.5	30.4	29.9	30.0	29.8	30.1	30.6	31.5	32.0	31.7	0.620	0.119
cis n-3 PUFA[13]	6.13^{e}	6.42^{d}	6.57^{d}	8.16^{a}	7.77^{b}	7.71^{bc}	7.85^{ab}	7.72^{b}	7.39^{c}	6.60^{d}	6.47^{de}	6.28^{de}	0.243	<0.001
cis n-6 PUFA[14]	21.1	21.5	21.8	21.0	20.3	20.3	19.7	19.5	20.0	20.1	20.5	20.7	0.606	0.163
n-3:n-6 ratio	0.28^{ef}	0.28^{ef}	0.31^{ef}	0.42^{a}	0.40^{ac}	0.38^{c}	0.40^{a}	0.37^{c}	0.35^{d}	0.30^{e}	0.28^{f}	0.27^{ef}	0.029	<0.001
n-6:n-3 ratio	4.13^{ab}	4.02^{ab}	3.68^{c}	2.89^{g}	3.00^{fg}	3.01^{defg}	2.87^{g}	3.00^{efg}	3.23^{df}	3.78^{bc}	3.96^{ab}	4.12^{a}	0.119	<0.001
EPA+DHA	0.47^{h}	0.52^{g}	0.51^{g}	0.59^{de}	0.62^{bc}	0.56^{f}	0.67^{a}	0.62^{bc}	0.65^{b}	0.61^{cd}	0.58^{ef}	0.56^{ef}	0.067	<0.001
*trans*FA[15]	33.9^{fgh}	35.8^{f}	38.6d	46.9^{a}	45.3^{b}	41.3^{c}	40.6^{c}	38.2^{d}	38.2^{d}	34.2^{fg}	32.7^{h}	33.5^{gh}	0.86	<0.001
*trans*FA (exc. VA)	22.7^{ef}	24.4^{d}	26.4^{c}	27.6^{a}	26.2^{b}	25.2^{cd}	25.1^{cd}	24.5^{d}	24.7^{d}	22.4^{ef}	21.7^{f}	23.0^{e}	0.56	<0.001
Human health related indices														
AI[16]	2.65^{c}	2.56^{d}	2.42^{e}	2.18^{g}	2.25^{f}	2.27^{f}	2.37^{e}	2.42^{e}	2.45^{de}	2.78^{b}	2.88^{a}	2.82^{ab}	0.041	<0.001
TI[17]	3.04^{c}	2.97^{d}	2.83^{e}	2.50^{i}	2.60^{h}	2.64^{gh}	2.70^{fg}	2.76^{ef}	2.80^{e}	3.10^{bc}	3.19^{a}	3.16^{ba}	0.036	<0.001
HH[18]	0.50^{f}	0.51^{e}	0.54^{cd}	0.60^{a}	0.58^{b}	0.58^{ab}	0.55^{c}	0.54^{cd}	0.53^{de}	0.47^{g}	0.45^{h}	0.46^{gh}	0.009	<0.001
Δ^9-desaturase activity indicators														
Δ^9I[19]	0.27^{e}	0.27^{d}	0.28^{c}	0.29^{a}	0.29^{b}	0.29^{ab}	0.28^{c}	0.28^{c}	0.28^{cd}	0.26^{f}	0.25^{g}	0.26^{fg}	0.027	<0.001
C14:1:C14:0	0.02^{f}	0.02^{e}	0.02^{ef}	0.02^{cd}	0.02^{cd}	0.02^{b}	0.02^{a}	0.02^{bc}	0.02^{de}	0.02^{g}	0.02^{h}	0.02^{gh}	0.002	<0.001
C16:1:C16:0	0.03^{fg}	0.03^{defg}	0.03^{ce}	0.03^{a}	0.03^{a}	0.03^{a}	0.03^{b}	0.03^{b}	0.03^{b}	0.03^{cdf}	0.03^{efg}	0.03^{g}	0.003	0.003
OA:C18:0	1.92^{bcd}	1.90^{cd}	1.88^{e}	1.93^{bcd}	1.94^{bcd}	1.94^{bcd}	1.93^{cde}	1.99^{a}	1.99^{a}	1.96^{ab}	1.94^{abc}	1.89^{de}	0.183	<0.001
RA:VA	0.17^{bc}	0.17^{bc}	0.16^{de}	0.15^{f}	0.16^{e}	0.16^{cd}	0.16^{bcd}	0.18^{a}	0.18^{a}	0.17^{b}	0.17^{bc}	0.17^{bcd}	0.015	<0.001

[1] n is the number of records used to calculate means.

[2] Significances were declared at $P < 0.05$.

[3]SFA: C4:0, C5:0, C6:0, C7:0, C8:0, C9:0, C10:0, C11:0, C12:0, C13:0, C14:0, C15:0, C16:0, C17:0, C18:0, C20:0, C22:0, C24:0.

[4]MUFA:C10:1 *c*9,C11:1 *c*10,C12:1 *c*9,C13:1,C14:1 *t*9,C15:1 *c*9,C16:1 *t*6-8,C16:1 *t*9,C16:1 *t*11+*t*12+*t*13, C16:1 (co-elutes with C17:0 anteiso, C16:1 *c*11,C16:1 *c*13, C17:1 *t*10, C17:1 *c*9, C18:1 *t*4, C18:1 *t*5, C18:1 *t*6-8, C18:1 *t*9, C18:1 *t*10, C18:1 *t*11 (VA), C18:1 *c*6+*t*12, C18:1 *c*9 (OA) ,C18:1 *t*15, C18:1 *c*11, C18:1 *c*12, C18:1 *c*13, C18:1 *c*14 (co-elutes with C18:1

*t*6), C18:1 *c*15 (co-elutes with C19:0), C18:1 *c*16, C19:1, C20:1 *c*8, C20:1 *c*11, C22:1 *c*13, C24:1 *c*15.

[5]PUFA: C18:2 *t*11*t*15, C18:2 *t*9*t*12, C18:2 *c*9*t*13, C18:2 *c*10*t*14, C18:2 *c*9*t*14, C18:2 *c*9*t*12, C18:2 *t*11*c*15, C18:2 *t*9*c*12, C18:2 *c*9*c*12 (LA), C18:2 *t*12*c*15, C18:3 *c*6*c*9*c*12, C18:3 *c*9*c*12*c*15 (ALNA), 18:2 *c*9*t*11 conjugated (RA) (co-elutes with C18:2 *t*7*c*9 + *t*8*c*10 + *t*6*c*8), other conjugated FA of unknown isomerisms of C18:2, C20:2 *c*11*c*14, C20:3 *c*8*c*11*c*14, C20:3 *c*11*c*14*c*17, C20:4 *c*5*c*8*c*11*c*14, C22:2 *c*13*c*16, C20:5 *c*5*c*8*c*11*c*14*c*17 (EPA), C22:3 *c*13*c*16*c*19, C22:4 *c*7*c*10*c*16*c*19, C22:5 *c*7*c*10*c*13*c*16*c*19 (DPA), C22:6 *c*4*c*7*c*10*c*13*c*16*c*19 (DHA).

[6]*cis*MUFA: C10:1 *c*9, C11:1 *c*10, C12:1 *c*9, C13:1 *c*9, C14:1 *c*9, C16:2 *c*9 (co-elutes with C17:0 anteiso), C16:1 *c*11, C16:1 *c*13, C17:1 *c*9, C18:1 *c*6 (co-elutes with C18:1 *t*16), C18:1 *c*9 (OA), C18:1 *c*11, C18:1 *c*12, C18:1 *c*13, C18:1 *c*14 (co-elutes with C18:1 *t*6), C18:1 *c*15 (co-elutes with C19:0), C18:1 *c*16, *c*19:1 *c*9, C20:1 *c*8, C20:1 *c*11, C22:1 *c*13, C24:1 *c*15.

[7]*trans*MUFA:C14:1 *t*9, C16:1 *t*6+*t*7+*t*8, C16:1 *t*9, C16:1 *t*11+*t*12+*t*13, C17:1 *t*10, C18:1 *t*4, C18:1 *t*5, C18:1 *t*6+*t*7+*t*8, C18:1 *t*9, C18:1 *t*10, C18:1 *t*11 (VA), C18:1 *t*12 (co-elutes with C18:1 *c*6), C18:1 *t*15, C18:1 *t*16 (co-elutes with C18:1 *c*14).

[8]*cis*PUFA:C18:2 *c*9*c*12 (LA), C18:3 *c*6*c*9*c*12, C18:3 *c*9*c*12*c*15 (ALNA), 20:2 *c*11*c*14, C20:3 *c*8*c*11*c*14, C20:3 *c*11*c*14*c*17, C20:4 *c*5*c*8*c*11*c*14, C22:3 *c*13*c*16, C20:5 *c*5*c*8*c*11*c*14*c*17 (EPA), C22:3 *c*13*c*16*c*19, C22:4 *c*7*c*10*c*13*c*16, C22:5 *c*7*c*10*c*13*c*16*c*19 (DPA), C22:6 *c*4*c*7*c*10*c*13*c*16*c*19 (DHA).

[9]*trans*PUFA: C18:2 *t*11*t*15, C18:2 *t*9*t*12.

[10]*cis,trans+trans,cis* PUFA: C18:2 *c*9*t*13, C18:2 *c*10*t*14, C18:2 *c*9*t*14, C18:2 *c*9*t*12, C18:2 *t*11*c*15, C18:2 *t*9*c*12, C18:2 *t*12*c*15, C18:2 *c*9*t*11 (RA) (co-elutes with C18:2 *t*7*c*9 + *t*8*c*10 + *t*6*c*8), other conjugated FA of unknown isomerism (CLA other a-h).

[11]omega-3 PUFA (n-3): C18:2 *t*11*t*15, C18:2 *t*11*c*15, C18:2 *t*12*c*15, C18:3 *c*9*c*12*c*15, C20:3 *c*11*c*14*c*17, C20:5 *c*5*c*8*c*11*c*14*c*17 (EPA), C22:3 *c*13*c*16*c*19, C22:5 *c*7*c*10*c*13*c*16*c*19 (DPA), C22:6 *c*4*c*7*c*10*c*13*c*16*c*19.

[12]omega-6 PUFA(n-6): C18:2 *t*9*t*12, C18:2 *c*9*t*12, C18:2 *t*9*c*12, C18:2 *c*9*c*12 (LA), C18:3 *c*6*c*9*c*12, C20:2 *c*11*c*14, C20:3 *c*8*c*11*c*14, C20:4 *c*5*c*8*c*11*c*14, C22:2 *c*13*c*16, C22:4 *c*7*c*10*c*13*c*16.

[13]*Cis* n-3 PUFA: C18:3 *c*9*c*12*c*15, C20:3 *c*11 *c*14 C17, C20:5 *c*5*c*8*c*11*c*14*c*17, C22:3 *c*13C16C19, C22:5 *c*7*c*10*c*13C16C19, C22:6 *c*4*c*7*c*10*c*13C16C19.

[14]*Cis*n-6 PUFA: C18:2 *c*9*c*12, C18:3 *c*6*c*9*c*12, C20:2 *c*11*c*14, C20:4 *c*5*c*8*c*11*c*14, C22:2 *c*13*c*16.

[15]*trans*FA: C14:1 *t*9, C16:1 *t*6+*t*7+*t*8, C16:1 *t*9, C16:1 *t*11+*t*12+*t*13, C17:1 *t*10, C18:1 *t*4, C18:1 *t*5, C18:1 *t*6+*t*7+*t*8, C18:1 *t*9, C18:1 *t*10, C18:1 *t*11 (VA), C18:1 *t*12 (co-elutes with C18:1 *c*6), C18:1 *t*15, C18:1 *t*16 (co-elutes with C18:1 *c*14), C18:2 *t*11*t*15, C18:2 *t*9*t*12.

[16]Atherogenicity index = (C12:0 + (4 × C14:0) + C16:0) / (MUFA + PUFA), as described in Średnicka-Tober, *et al.* [3].

[17]Thrombogenicity index= (C14:0 + C16:0 + C18:0) / (0.5 × MUFA) + (0.5 × n-6) + (3×n-3) + (n-3:n-6) as described in Średnicka-Tober, et al. [3].

[18]Hypocholesterolemic to hypercholesterolemic ratio = (C18:1 *c*9 + total PUFA) / (C12:0 + C14:0 + C16:0) as described in Mierlita [4].

[19]Δ9-desaturase activity index= (*c*9 C14:1 + *c*9 C16:1 + OA + RA) / (*c*9 C14:1 + *c*9 C16:1 + OA + RA + C14:0 + C16:0 + C18:0 + VA) as described in Kay, *et al.* [5].

Table S6. Means ± SE and P-values for the FA profile of milk from 67 farms with two differing production systems (Conventional and organic) in Southern England between January and December (2019)

	Month													
	January n=65[1]	February n=67[1]	March n=67[1]	April n=67[1]	May n=67[1]	June n=67[1]	July n=67[1]	August n=67[1]	September n=67[1]	October n=67[1]	November n=67[1]	December n=65[1]	SE	P-Value[2]
C4:0	26.5^{bc}	26.6^{bc}	27.2^{a}	26.6^{b}	26.4^{bc}	26.0^{cd}	27.5^{a}	27.1^{a}	26.8^{b}	25.4^{e}	25.0^{f}	25.8^{de}	2.32	<0.001
C5:0	0.27^{b}	0.26^{bd}	0.25^{cde}	0.26^{bcd}	0.24^{fg}	0.20^{h}	0.24^{g}	0.25^{def}	0.24^{efg}	0.26^{b}	0.29^{a}	0.28^{a}	0.022	<0.001
C6:0	18.9^{bc}	18.8^{bc}	18.8^{bc}	18.6^{c}	18.2^{d}	17.9^{e}	18.9^{bc}	18.8^{bc}	18.9^{bc}	19.1^{ab}	19.0^{b}	19.3^{a}	1.63	<0.001
C7:0	0.25^{de}	0.24^{ef}	0.23^{efg}	0.25^{cd}	0.22^{g}	0.17^{i}	0.20^{h}	0.21^{g}	0.22^{fg}	0.27^{c}	0.31^{a}	0.30^{b}	0.021	<0.001
C8:0	11.8^{bcdef}	11.7^{bcdef}	11.6^{cdef}	11.7^{bd}	11.4^{ef}	11.1^{g}	11.5^{f}	11.5^{def}	11.7^{bce}	12.3^{a}	12.4^{a}	12.4^{a}	1.04	<0.001
C9:0	0.32^{cd}	0.32^{cd}	0.29^{ef}	0.32^{c}	0.29^{de}	0.23^{g}	0.25^{g}	0.27^{f}	0.30^{de}	0.36^{b}	0.39^{a}	0.37^{b}	0.029	<0.001
C10:0	27.8^{c}	27.4^{cd}	26.9^{de}	27.4^{c}	27.0^{cd}	25.7^{e}	25.9^{e}	26.0^{e}	26.7^{cd}	28.9^{b}	29.6^{a}	29.5^{ab}	2.49	<0.001
C10:1 *c*9	2.77^{cd}	2.70^{cd}	2.55^{e}	2.49^{e}	2.47^{e}	2.44^{e}	2.64^{d}	2.73^{c}	2.79^{c}	3.04^{ab}	3.06^{a}	2.99^{b}	0.243	<0.001
C11:0	0.56^{defg}	0.60^{def}	0.53^{g}	0.58^{df}	0.54^{efg}	0.42^{hi}	0.42^{i}	0.45^{h}	0.54^{fg}	0.68^{c}	0.77^{a}	0.74^{b}	0.054	<0.001
C11:1 *c*10	0.02^{fg}	0.02^{ef}	0.01^{gh}	0.02^{f}	0.02^{de}	0.01^{h}	0.02^{fg}	0.02^{ef}	0.02^{d}	0.03^{b}	0.03^{a}	0.02^{c}	0.001	<0.001
C12:0	35.0^{c}	34.5^{c}	33.6^{def}	34.0^{c}	33.2^{cde}	31.7^{g}	32.2^{fg}	32.6^{efg}	33.6^{cd}	36.3^{b}	37.2^{a}	36.8^{ab}	3.16	<0.001
C13:0 iso	0.19^{h}	0.24^{fg}	0.23^{g}	0.27^{de}	0.28^{bc}	0.27^{cd}	0.31^{a}	0.29^{b}	0.28^{bcd}	0.25^{ef}	0.24^{g}	0.25^{f}	0.031	<0.001
C13:0 anteiso	0.08^{d}	0.11^{bc}	0.10^{bc}	0.10^{c}	0.12^{a}	0.11^{ab}	0.11^{ab}	0.11^{abc}	0.07^{e}	0.08^{d}	0.11^{ab}	0.11^{abc}	0.012	<0.001
C12:1 *c*9	0.84^{def}	0.86^{cde}	0.79^{g}	0.81^{de}	0.82^{de}	0.78^{fg}	0.81^{efg}	0.85^{cd}	0.88^{c}	0.99^{ab}	1.02^{a}	0.97^{b}	0.082	<0.001
C13:0	1.00^{de}	1.00^{d}	0.91^{gh}	0.93^{ef}	0.91^{fg}	0.79^{i}	0.83^{i}	0.84^{hi}	0.91^{fg}	1.07^{c}	1.19^{a}	1.14^{b}	0.088	<0.001
c14:0 iso	0.91^{bcd}	0.93^{ab}	0.91^{bcd}	0.84^{f}	0.85^{ef}	0.89^{cd}	0.96^{a}	0.91^{bc}	0.89^{de}	0.82^{f}	0.82^{f}	0.84^{f}	0.100	<0.001
C13:1	0.05^{f}	0.06^{cd}	0.04^{h}	0.04^{h}	0.05^{def}	0.04^{g}	0.05^{ef}	0.05^{cdf}	0.06^{c}	0.06b	0.07^{a}	0.07^{b}	0.005	<0.001
C14:0	112^{cd}	110^{d}	107^{e}	105^{g}	106^{efg}	104^{fg}	106^{eg}	106^{efg}	107^{e}	113^{bc}	115^{a}	114^{ab}	9.7	<0.001
C14:1 *t*9	2.17^{efg}	2.22^{de}	2.17^{fg}	2.24^{e}	2.28^{cd}	2.34b	2.50^{a}	2.34^{bc}	2.24^{def}	2.11^{g}	2.07^{h}	2.09^{gh}	0.249	<0.001
C15:0	4.32^{de}	4.30^{de}	4.10^{g}	4.56^{c}	4.91^{a}	4.78^{b}	4.88^{ab}	4.55^{c}	4.35^{d}	4.18^{eg}	4.11^{g}	4.08^{g}	0.472	<0.001
C15:1 *c*9	9.76^{cd}	9.36^{e}	8.72^{f}	8.46^{g}	8.72^{f}	8.79^{f}	9.32^{de}	9.76^{c}	9.74^{c}	10.6^{a}	10.7^{a}	10.2^{b}	0.852	<0.001
C15:0 anteiso	11.7^{ab}	11.2^{c}	10.5^{f}	9.99^{g}	10.3^{f}	10.0^{g}	10.5^{def}	10.4^{ef}	10.7^{df}	11.5^{bc}	12.1^{a}	11.7^{ab}	1.012	<0.001
C16:0 iso	2.13^{bc}	2.18^{a}	2.20^{a}	2.01^{e}	2.10^{cd}	2.16^{ab}	2.17^{ab}	2.02^{de}	1.90^{hf}	1.86^{gh}	1.86^{h}	1.90^{fgh}	0.222	<0.001
C16:0	321^{c}	315^{d}	306^{ef}	287^{i}	295^{h}	300^{g}	306^{f}	314^{de}	313^{de}	330^{ab}	334^{a}	327^{d}	27.9	<0.001
C16:1 *t*6-8	0.29^{bc}	0.32^{a}	0.33^{a}	0.32^{a}	0.23^{e}	0.27^{d}	0.28^{bc}	0.29^{bc}	0.30^{b}	0.28^{cd}	0.33^{a}	0.28^{bcd}	0.027	<0.001
C16:1 *t*9	0.08^{c}	0.11^{a}	0.09^{bc}	0.08^{c}	0.06^{f}	0.05^{g}	0.08^{c}	0.07^{ef}	0.08^{cde}	0.07^{def}	0.08^{ce}	0.10^{ab}	0.009	<0.001
C17:0 iso	3.30^{e}	3.37^{e}	3.35^{e}	4.12^{b}	4.36^{a}	4.31^{a}	4.13^{b}	3.90^{c}	3.73^{c}	3.27^{d}	3.22^{e}	3.22^{e}	0.421	<0.001

C16:1 *t*11/13	1.55^{i}	1.81^{g}	1.88^{f}	2.28^{b}	2.35^{a}	2.27^{b}	2.20^{c}	2.08^{c}	1.98^{e}	1.75^{e}	1.68^{g}	1.68^{h}	0.215	<0.001
C16:1 + C17:0 anteiso	17.8^{de}	17.6^{de}	17.4^{e}	17.7^{d}	18.3^{bc}	18.5^{abc}	18.3^{bc}	18.8^{a}	18.6^{ab}	18.7^{a}	18.6^{ab}	17.8^{cd}	1.75	<0.001
C16:1 *c*11	0.90^{h}	1.04^{abcde}	1.05^{cde}	1.06^{de}	0.93^{gh}	0.96^{fgh}	1.07^{bcde}	1.14^{bcd}	1.16^{ac}	1.02^{efh}	1.00^{efgh}	0.96^{efgh}	0.124	<0.001
C16:1 *c*13	1.29^{c}	1.19^{de}	1.06^{gh}	1.07^{fgh}	1.33^{abc}	1.15^{ef}	1.02^{h}	1.09^{efg}	1.12^{efg}	1.35^{ac}	1.40^{a}	1.25^{bcd}	0.126	<0.001
C17:0	5.32^{bc}	5.40^{bc}	5.38^{bc}	5.31^{bc}	4.88^{d}	5.27^{bc}	5.57^{a}	5.40^{b}	5.34^{bc}	5.26^{c}	5.32^{bc}	5.32^{bc}	0.521	<0.001
C17:1 *t*10	0.48^{d}	0.52^{cb}	0.57^{a}	0.58^{a}	0.44^{e}	0.49^{cd}	0.57^{a}	0.53^{b}	0.57^{a}	0.53^{b}	0.51^{cd}	0.52^{bc}	0.049	<0.001
C18:0 iso	0.47^{bc}	0.31^{g}	0.44^{ef}	0.46^{cb}	0.41^{f}	0.42^{ef}	0.55^{a}	0.45^{ef}	0.50^{b}	0.46^{ce}	0.49^{b}	0.49^{b}	0.052	<0.001
C17:0 *c*9	2.02^{efg}	2.02^{fg}	2.01^{fg}	2.11^{cde}	2.10^{deg}	2.25^{a}	2.21^{ab}	2.19^{abc}	2.15^{bcd}	1.99^{g}	2.06^{ef}	2.01^{efg}	0.223	<0.001
C18:0	98.5^{e}	101^{cd}	106^{ab}	106^{ab}	105^{ab}	107^{a}	103^{bc}	99.9^{de}	99.1^{de}	92.8^{fg}	91.4^{g}	95.2^{f}	10.05	<0.001
C18:1 *t*4	0.15^{cd}	0.19^{a}	0.18^{b}	0.17^{b}	0.13^{e}	0.14^{de}	0.18^{ab}	0.17^{bc}	0.18^{ab}	0.18^{bc}	0.18^{ab}	0.18^{ab}	0.019	<0.001
C18:1 *t*5	0.14^{f}	0.16^{cb}	0.17^{b}	0.18^{a}	0.11^{g}	0.11^{g}	0.16^{cdf}	0.15^{ef}	0.15^{def}	0.15^{f}	0.15^{f}	0.15^{f}	0.015	<0.001
C18:1 *t*6-8	2.91^{efghi}	3.10^{cdf}	3.26^{ab}	3.30^{a}	3.18^{bc}	2.92^{fghi}	2.99^{defg}	2.88^{ghi}	2.93^{fgh}	2.75^{i}	2.76^{hi}	2.82^{hi}	0.340	<0.001
C18:1 *t*9	2.35^{abc}	2.33^{bc}	2.32^{bc}	2.38^{ab}	2.48^{a}	2.30^{bc}	2.11^{ef}	2.20^{cdef}	2.21^{cde}	2.10^{f}	2.14^{def}	2.25^{ce}	0.265	<0.001
C18:1 *t*10	4.59^{cd}	5.25^{ab}	5.61^{ab}	5.68^{ab}	5.71^{a}	5.50^{a}	5.14^{bc}	5.36^{ab}	5.55^{ab}	4.39^{d}	3.72^{e}	4.20^{de}	0.718	<0.001
C18:1 *t*11	11.2^{def}	11.4^{def}	12.2^{de}	19.3^{a}	19.0^{a}	16.1^{b}	15.4^{b}	13.7^{c}	13.5^{c}	11.8^{d}	11.0^{d}	10.6^{f}	1.88	<0.001
C18:1 *c*6/*t*12	4.42^{c}	4.70^{b}	5.16^{a}	4.84^{b}	4.40^{c}	4.11^{df}	3.95^{ef}	3.89^{ef}	4.27^{cd}	3.92^{f}	3.91^{f}	4.12^{de}	0.465	<0.001
C18:1 *c*9	189^{h}	191^{gh}	197^{def}	204^{ab}	202^{ecb}	206^{a}	198^{cdef}	198^{ef}	196^{fg}	181^{i}	177^{j}	179^{ij}	19.0	<0.001
C18:1 *t*15	2.75^{g}	2.99^{f}	3.96^{ab}	4.11^{a}	3.30^{de}	3.28^{d}	3.72^{bc}	3.27^{de}	3.21^{def}	3.26^{def}	3.10^{ef}	3.74^{c}	0.356	<0.001
C18:1 *c*11	5.69^{fg}	5.97^{cde}	6.50^{a}	6.57^{a}	5.89^{bcdf}	6.08^{c}	6.13^{bc}	5.67^{efg}	5.90^{cdf}	5.49^{g}	5.24^{h}	5.72^{defg}	0.699	<0.001
C18:1 *c*12	2.58^{bc}	2.63^{b}	2.75^{a}	2.32^{deg}	2.17^{fg}	2.18^{fg}	2.00^{h}	1.99^{h}	2.17^{g}	2.20^{efg}	2.36^{cd}	2.42^{cd}	0.283	<0.001
C18:1 *c*13	0.56^{e}	0.71^{c}	0.76^{b}	0.84^{a}	0.58^{e}	0.51^{fg}	0.70^{c}	0.77^{b}	0.82^{a}	0.66^{d}	0.49^{g}	0.55^{ef}	0.059	<0.001
C18:1 *t*6 + C18:1 *c*14	3.34^{defg}	3.30^{efgh}	3.56^{c}	3.93^{a}	3.71^{b}	3.55^{c}	3.51^{cd}	3.44^{cde}	3.40^{def}	3.21^{h}	3.22^{gh}	3.27^{fgh}	0.354	<0.001
C19:0 + C18:1 *c*15	1.41^{def}	1.44^{efg}	1.48^{ef}	1.58^{c}	1.67^{b}	1.75^{a}	1.69^{ab}	1.58^{cd}	1.46^{efg}	1.39^{g}	1.43^{fg}	1.49^{cde}	0.174	<0.001
C18:2 *t*11*t*15	0.18^{f}	0.20^{f}	0.22^{ef}	0.45^{a}	0.41^{b}	0.33^{c}	0.29^{d}	0.29^{d}	0.25^{e}	0.20^{f}	0.18^{f}	0.18^{f}	0.045	<0.001
C18:2 *t*9*t*12	0.04^{fgh}	0.04^{fgh}	0.04^{cdefg}	0.05^{b}	0.06^{b}	0.04^{bcdef}	0.05^{bcf}	0.04^{defg}	0.04^{bcdef}	0.04^{efg}	0.03^{h}	0.03^{gh}	0.006	<0.001
C18:2 *c*8*c*13	2.01^{efg}	2.12^{cde}	2.19^{bcd}	2.58^{a}	2.59^{a}	2.30^{b}	2.06^{def}	2.21^{bc}	2.23^{bc}	2.01^{efg}	1.98^{fg}	1.89^{g}	0.249	<0.001
C18:2 *c*10*t*14	1.15^{bcd}	1.24^{a}	1.15^{cdf}	1.08^{ef}	1.13^{cd}	1.09^{def}	1.09^{def}	1.06^{f}	0.86^{g}	1.10^{def}	1.24^{ab}	1.23^{abc}	0.121	<0.001
C18:2 *c*9*c*14	1.22^{defg}	1.26^{bcde}	1.28^{bc}	1.39^{a}	1.36^{a}	1.30^{b}	1.24^{cdef}	1.30^{b}	1.29^{bcd}	1.21^{efg}	1.20^{gf}	1.18^{g}	0.137	<0.001
C18:2 *c*9*t*12	0.63^{cd}	0.63^{cde}	0.66^{b}	0.70^{a}	0.66^{b}	0.63^{d}	0.60^{efg}	0.60^{cdefg}	0.60^{def}	0.58^{g}	0.59^{fg}	0.60^{defg}	0.067	<0.001
C18:1 *c*16	0.28^{cd}	0.27^{de}	0.29^{cd}	0.39^{b}	0.47^{a}	0.39^{b}	0.29^{c}	0.27^{cd}	0.29^{cd}	0.25^{e}	0.20^{f}	0.25^{e}	0.042	<0.001
C18:1 *t*11*c*15	1.25^{ef}	1.24^{ef}	1.31^{ef}	2.46^{a}	2.22^{b}	1.87^{cd}	1.99^{c}	1.87^{cd}	1.80^{d}	1.41^{e}	1.18^{f}	1.09^{f}	0.245	<0.001
C18:2 *t*9*c*12	0.20^{a}	0.19^{b}	0.18^{b}	0.09^{efg}	0.06h	0.09^{ef}	0.07^{gh}	0.07^{fgh}	0.09^{e}	0.14^{dc}	0.12^{d}	0.14^{c}	0.016	<0.001
C18:2 *c*9*c*12	18.4^{cdef}	18.8^{be}	19.2^{a}	18.4^{bce}	17.8^{def}	17.8^{def}	17.0^{h}	17.0^{gh}	17.5^{fh}	17.5^{fgh}	17.7^{efh}	17.9^{efgh}	1.986	<0.001

C18:2 *t*12*c*15	0.99^{b}	0.78^{efg}	1.15^{a}	0.93^{cd}	0.87^{de}	0.99^{cb}	1.14^{a}	0.97^{bc}	0.74^{g}	0.78^{g}	0.78^{fg}	0.84^{def}	0.107	<0.001
C19:1	0.18^{cd}	0.23^{b}	0.26^{a}	0.25^{a}	0.21^{b}	0.21^{b}	0.19^{c}	0.17^{d}	0.13^{ef}	0.15^{e}	0.13^{f}	0.16^{d}	0.024	<0.001
C20:0	1.75^{a}	1.76^{a}	1.68^{b}	1.54^{e}	1.45^{f}	1.62^{dc}	1.62^{cd}	1.57^{e}	1.59^{de}	1.59^{de}	1.68^{bc}	1.81^{a}	0.168	<0.001
C18:3 *c*6*c*9*c*12	0.21^{bc}	0.18^{gh}	0.19^{ef}	0.20^{bcdf}	0.17^{h}	0.19^{cdef}	0.21^{b}	0.19^{defg}	0.19^{fg}	0.19^{defg}	0.22^{b}	0.24^{a}	0.021	<0.001
C20:1 *c*8	1.19^{bc}	1.22^{a}	1.22^{ab}	1.10^{efg}	1.03^{h}	1.08^{g}	1.13^{def}	1.09^{g}	1.09^{g}	1.09^{gf}	1.16^{dc}	1.13^{cde}	0.112	<0.001
C20:1 *c*11	0.57^{cde}	0.65^{ab}	0.64^{a}	0.59^{bcd}	0.66^{abc}	0.51^{e}	0.36^{f}	0.38^{f}	0.55^{de}	0.51^{e}	0.49^{e}	0.67^{a}	0.069	<0.001
C18:3 *c*9c12*c*15	4.68^{ef}	4.92^{df}	5.11^{d}	6.60^{a}	6.08^{b}	6.06^{b}	6.01^{b}	6.01^{b}	5.64^{c}	4.91^{def}	4.86^{def}	4.66^{f}	0.685	<0.001
CLA c9 *t*11 + *t*7 *c*9 + *t*8 *c*10 + *t*6 *t*8	5.59^{de}	5.71^{de}	5.80^{de}	8.44^{a}	8.80^{a}	7.76^{b}	7.56^{bc}	7.27^{c}	7.17^{c}	5.95^{d}	5.44^{e}	5.24^{e}	0.897	<0.001
CLA other a	0.25^{def}	0.27^{abc}	0.24^{ef}	0.21^{g}	0.24^{f}	0.26^{bc}	0.28^{a}	0.26^{bcd}	0.26^{bcd}	0.25^{cde}	0.25^{cde}	0.26^{cde}	0.028	<0.001
CLA other b	0.21^{ghi}	0.24^{efhi}	0.23^{fghi}	0.34^{a}	0.28^{bde}	0.26^{defgh}	0.29^{be}	0.29^{b}	0.29^{b}	0.25^{defg}	0.21^{i}	0.21^{hi}	0.043	<0.001
CLA other c	0.15^{de}	0.16^{bcd}	0.13^{f}	0.16^{bcd}	0.15^{cd}	0.14^{ef}	0.18^{a}	0.17^{ab}	0.17^{abc}	0.15^{de}	0.16^{bcd}	0.15^{d}	0.017	<0.001
CLA other d	0.34^{ef}	0.36^{ef}	0.34^{ef}	0.49^{b}	0.51^{a}	0.43^{c}	0.43^{c}	0.42^{cd}	0.40^{d}	0.35^{ef}	0.36^{e}	0.33^{f}	0.047	<0.001
CLA other e	0.22^{abc}	0.22^{abc}	0.21^{defg}	0.22^{a}	0.22^{ab}	0.22^{ab}	0.22^{a}	0.21^{cde}	0.21^{bcd}	0.20^{gf}	0.20^{ef}	0.20^{g}	0.022	<0.001
CLA other f	0.05^{b}	0.06^{a}	0.06^{a}	0.04^{def}	0.05^{c}	0.05^{cde}	0.04^{f}	0.05^{cde}	0.04^{cdef}	0.04^{cdef}	0.04^{ef}	0.04^{cd}	0.006	<0.001
CLA other g	0.12^{deg}	0.12^{deg}	0.11^{fg}	0.20^{ab}	0.21^{a}	0.19^{cb}	0.20^{a}	0.19^{cb}	0.18^{c}	0.14^{d}	0.11^{efg}	0.10^{g}	0.025	<0.001
CLA other h	0.13^{c}	0.12^{c}	0.10^{d}	0.10^{d}	0.13^{c}	0.14^{b}	0.16^{a}	0.13^{c}	0.12^{c}	0.12^{c}	0.16^{a}	0.15^{a}	0.016	<0.001
C20:2 *c*11*c*14	0.20^{defg}	0.23^{a}	0.21^{bce}	0.17^{h}	0.18^{h}	0.19^{fg}	0.20^{cde}	0.19^{g}	0.19^{g}	0.20^{cdef}	0.20^{efg}	0.22^{ab}	0.021	<0.001
C22:0	0.69^{abcd}	0.71^{b}	0.59^{ef}	0.55^{g}	0.49^{h}	0.57^{fg}	0.74^{a}	0.69^{dc}	0.68^{d}	0.63^{d}	0.69^{bc}	0.72^{a}	0.071	<0.001
C20:3 *c*8*c*11*c*14	0.85^{abcd}	0.85^{ac}	0.83^{bcd}	0.80^{ef}	0.70^{h}	0.77^{g}	0.82^{dc}	0.79^{efg}	0.79^{gf}	0.80^{de}	0.85^{abc}	0.87^{abc}	0.083	<0.001
C22:1 *c*13	0.42^{cd}	0.40^{cd}	0.60^{a}	0.49^{cb}	0.18^{e}	0.16^{e}	0.15^{e}	0.13^{e}	0.15^{e}	0.22^{e}	0.32^{d}	0.55^{ab}	0.067	<0.001
C20:3 *c*11*c*14*c*17	0.08^{cd}	0.09^{bc}	0.07^{d}	0.09^{b}	0.08^{bcd}	0.09^{b}	0.12^{a}	0.10^{b}	0.10^{b}	0.09^{bc}	0.10^{b}	0.09^{b}	0.014	<0.001
C20:4 *c*5*c*8*c*11*c*14	1.07^{cdf}	1.08^{abcd}	1.08^{abcd}	1.08^{abcd}	0.97^{h}	1.01^{fg}	1.05^{d}	1.00^{fgh}	1.00^{gh}	1.05^{bcd}	1.10^{ac}	1.11^{ac}	0.105	<0.001
C22:2 *c*13*c*16	0.34^{fg}	0.35^{g}	0.30^{h}	0.37^{def}	0.47^{a}	0.42^{b}	0.41^{bc}	0.41^{b}	0.39^{cdf}	0.36^{efg}	0.36^{efg}	0.36^{defg}	0.049	<0.001
C20:5 *c*5*c*8*c*11*c*14*c*17 (EPA)	0.42^{h}	0.47^{g}	0.47^{g}	0.55^{cd}	0.57^{b}	0.52^{f}	0.60^{a}	0.57^{bc}	0.58^{b}	0.54^{d}	0.51^{e}	0.49^{fg}	0.061	<0.001
C24:0	0.37^{i}	0.41^{efh}	0.42^{bcde}	0.40^{bcde}	0.41^{f}	0.40^{h}	0.45^{a}	0.38^{i}	0.44^{abc}	0.42^{defg}	0.42^{cdef}	0.43^{abd}	0.046	<0.001
C24:1 *c*15	0.06^{d}	0.05^{e}	0.07^{c}	0.09^{b}	0.07^{c}	0.08^{b}	0.10^{a}	0.08^{b}	0.07^{cd}	0.06^{cd}	0.09^{b}	0.10^{a}	0.011	<0.001
C22:3 *c*13*c*16*c*19	0.11^{a}	0.11^{a}	0.11^{a}	0.09^{bc}	0.09^{cd}	0.08^{d}	0.09^{cd}	0.08^{cd}	0.08^{cd}	0.09^{cd}	0.10^{ab}	0.10^{a}	0.012	<0.001
C22:4 *c*7*c*10*c*13*c*16	0.07^{bcd}	0.06^{ef}	0.04^{g}	0.03^{h}	0.06^{de}	0.07^{cb}	0.08^{a}	0.07^{b}	0.06^{cde}	0.06^{f}	0.07^{b}	0.07^{b}	0.008	<0.001
C22:5 *c*7*c*10*c*13*c*16*c*19 (DPA)	0.79^{d}	0.79^{d}	0.76^{e}	0.79^{d}	0.90^{b}	0.91^{b}	0.96^{a}	0.91^{b}	0.92^{b}	0.90^{b}	0.87^{c}	0.84^{c}	0.099	<0.001
C22:6 *c*4 *c*7*c*10*c*13*c*16*c*19 (DHA)	0.05^{f}	0.06^{ef}	0.05^{gh}	0.04^{h}	0.05^{g}	0.04^{gh}	0.06^{ce}	0.06^{ef}	0.07^{ab}	0.07^{a}	0.07^{h}	0.07^{ab}	0.007	<0.001

[1]n is the number of records used to calculate means ± SE and P-values. Records with missing values were not included in the analysis

[2]Significances were declared at $P<0.05$. Means for season within a row with different lower-case letters are significantly different according to Fisher's Least Significant Difference test ($P<0.05$).

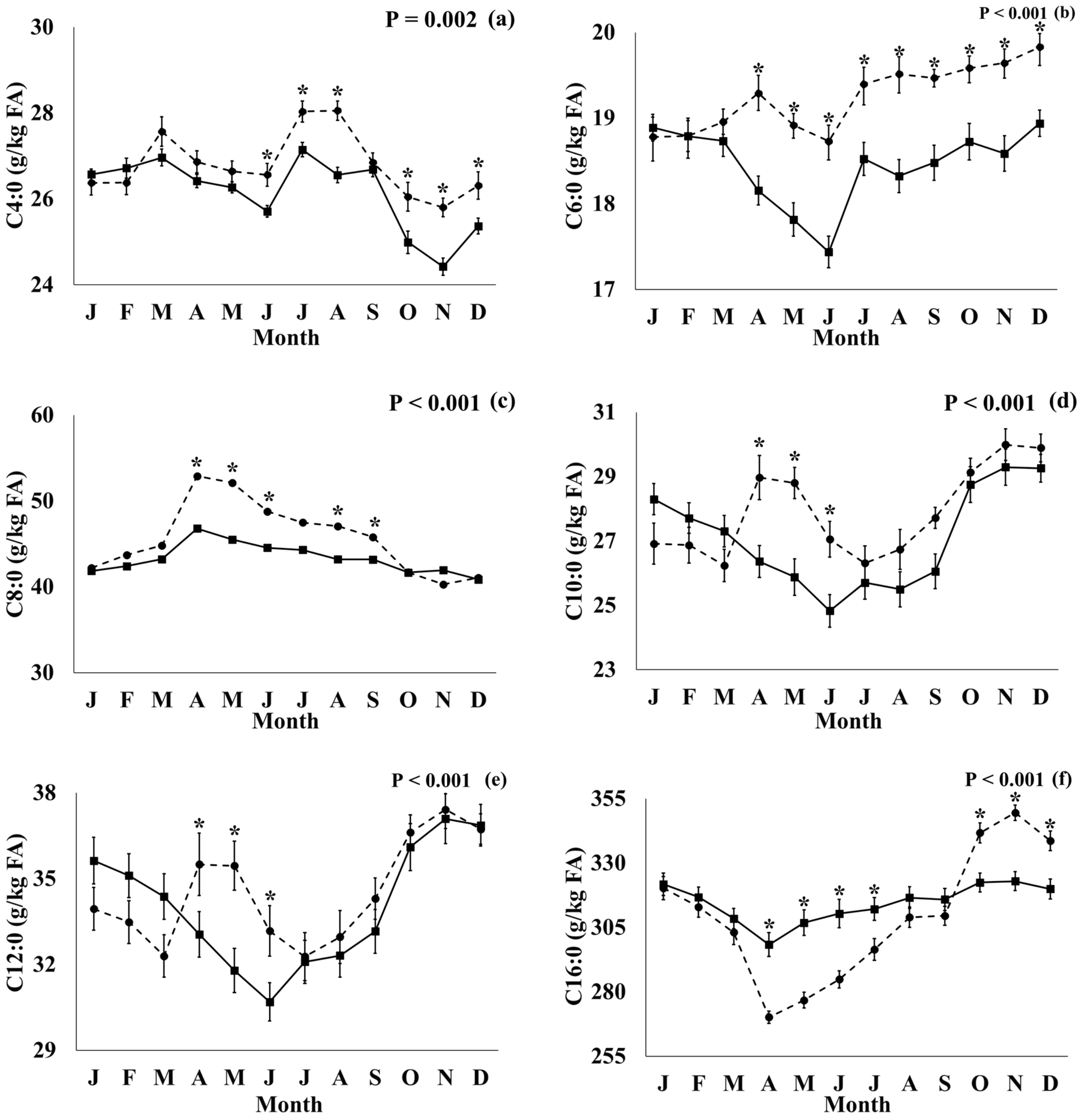

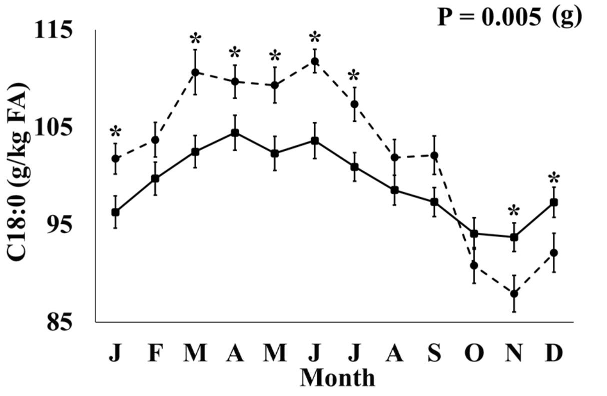

Figure S2: Individual saturated fatty acids not displayed in main text, where a significant ($p < 0.05$) interaction between production system and month was observed. Means were calculated from the measured values. The error bars represent the standard error of the means. *: significant difference between conventional and organic farms within the month ($p < 0.05$).

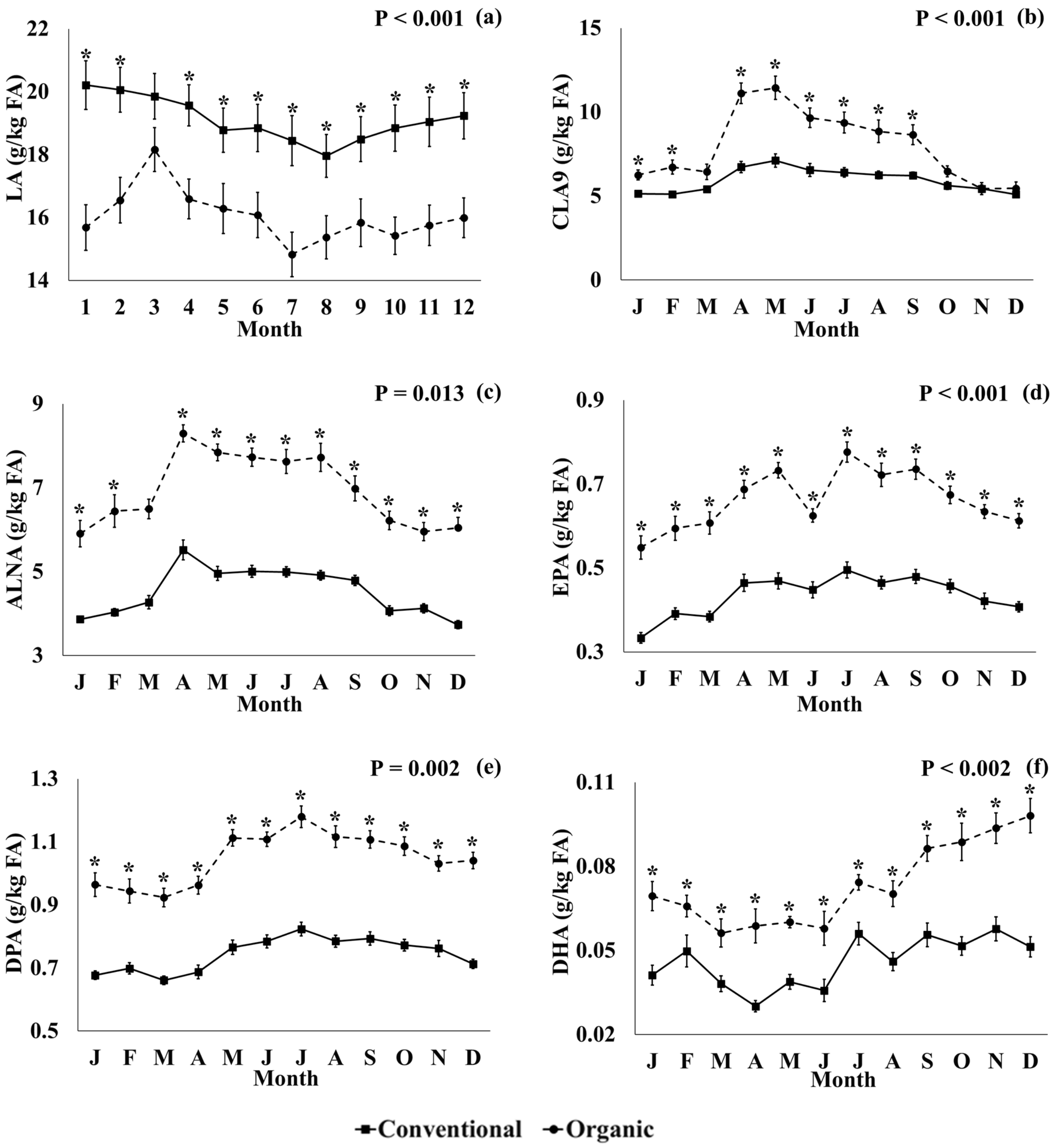

Figure S3: Individual polyunsaturated fatty acids not displayed in main text, where a significant ($p < 0.05$) interaction between production system and month was observed. Means were calculated from the measured values. The error bars represent the standard error of the means. *: significant difference between conventional and organic farms within the month ($p < 0.05$).

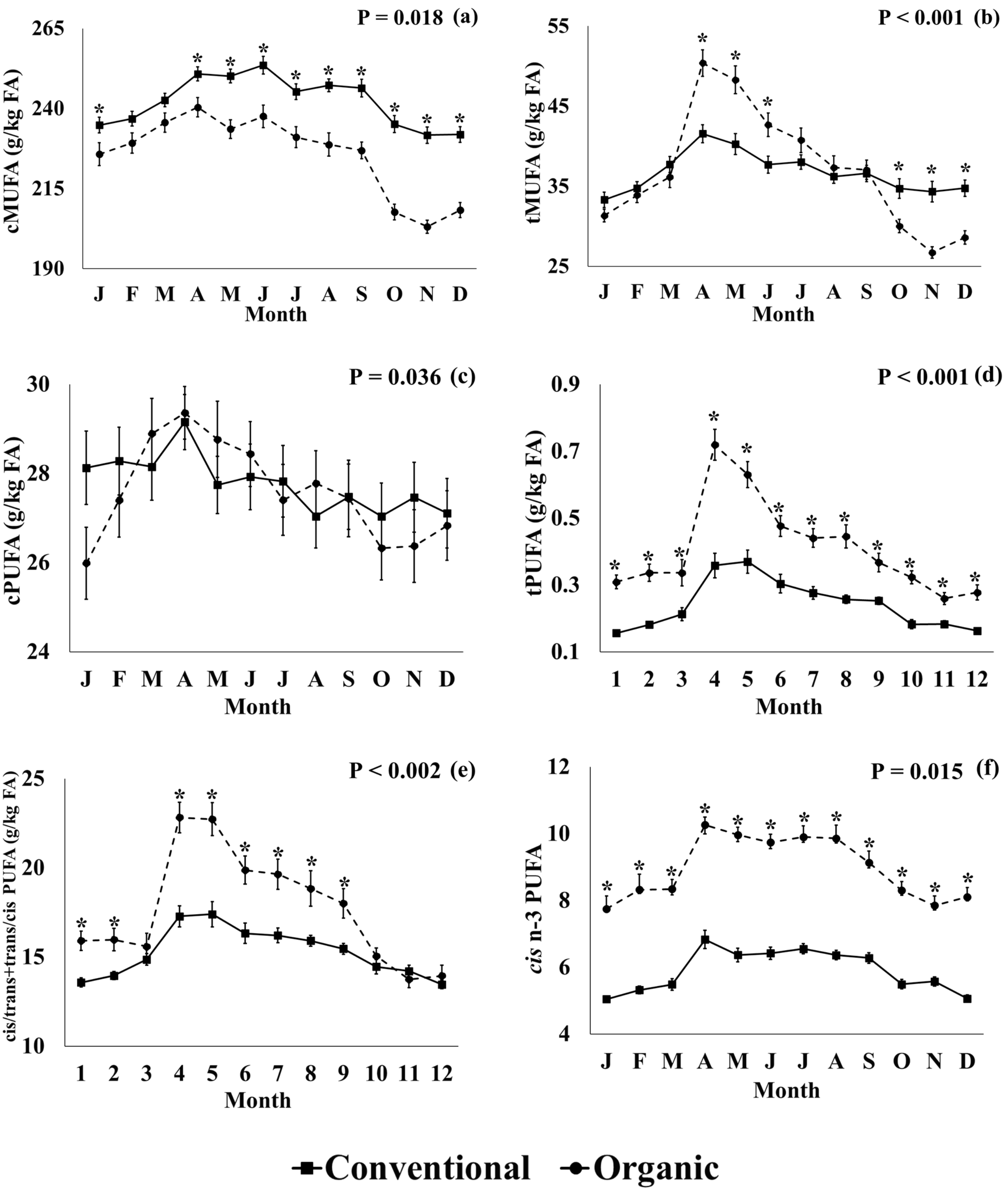

Figure S4: FA groups not displayed in main text, where a significant ($p < 0.05$) interaction between production system and month was observed. Means were calculated from the measured values. The error bars represent the standard error of the means. *: significant difference between conventional and organic farms within the month ($p < 0.05$).

References (supplementary material)

1. Stergiadis, S.; Bieber, A.; Chatzidimitriou, E.; Franceschin, E.; Isensee, A.; Rempelos, L.; Baranski, M.; Maurer, V.; Cozzi, G.; Bapst, B.; et al. Impact of US Brown Swiss genetics on milk quality from low-input herds in Switzerland: Interactions with season. *Food Chem.* **2018**, *251*, 93-102, doi:10.1016/j.foodchem.2018.01.077 .
2. Butler, G.; Nielsen, J.H.; Slots, T.; Seal, C.; Eyre, M.D.; Sanderson, R.; Leifert, C. Fatty acid and fat-soluble antioxidant concentrations in milk from high- and low-input conventional and organic systems: seasonal variation. *J. Sci. Food Agric.* **2008**, *88*, 1431-1441, doi:https://doi.org/10.1002/jsfa.3235.
3. Średnicka-Tober, D.; Barański, M.; Seal, C.; Sanderson, R.; Benbrook, C.; Steinshamn, H.; Gromadzka-Ostrowska, J.; Rembiałkowska, E.; Skwarło-Sońta, K.; Eyre, M.; et al. Composition differences between organic and conventional meat: a systematic literature review and meta-analysis. *Br. J. Nutr.* **2016**, *115*, 994-1011, doi:10.1017/S0007114515005073.
4. Mierlita, D. Effects of diets containing hemp seeds or hemp cake on fatty acid composition and oxidative stability of sheep milk. *S. Afr. J. Anim.* **2018**, *48*, 504-515, doi:10.4314/sajas.v48i3.11.
5. Kay, J.K.; Mackle, T.R.; Auldist, M.J.; Thomson, N.A.; Bauman, D.E. Endogenous Synthesis of cis-9, trans-11 Conjugated Linoleic Acid in Dairy Cows Fed Fresh Pasture. *Journal of Dairy Science* **2004**, *87*, 369-378, doi:10.3168/jds.S0022-0302(04)73176-8.
